# Antibacterial Compounds from Propolis of *Tetragonula laeviceps* and *Tetrigona melanoleuca* (Hymenoptera: Apidae) from Thailand

**DOI:** 10.1371/journal.pone.0126886

**Published:** 2015-05-18

**Authors:** Sirikarn Sanpa, Milena Popova, Vassya Bankova, Tawee Tunkasiri, Sukum Eitssayeam, Panuwan Chantawannakul

**Affiliations:** 1 The Graduate School, Chiang Mai University, Chiang Mai, Thailand; 2 Department of Biology, Faculty of Science, Chiang Mai University, Chiang Mai, Thailand; 3 Institute of Organic Chemistry with Centre of Phytochemistry, Bulgarian Academy of Sciences, Sofia, Bulgaria; 4 Department of Physics, Faculty of Science, Chiang Mai University, Chiang Mai, Thailand; University of British Columbia, CANADA

## Abstract

This study investigated the chemical composition and antimicrobial activity of propolis collected from two stingless bee species *Tetragonula laeviceps* and *Tetrigona melanoleuca* (Hymenoptera: Apidae). Six xanthones, one triterpene and one lignane were isolated from *Tetragonula laeviceps* propolis. Triterpenes were the main constituents in *T*. *melanoleuca* propolis. The ethanol extract and isolated compounds from *T*. *laeviceps* propolis showed a higher antibacterial activity than those of *T*. *melanoleuca* propolis as the constituent α-mangostin exhibited the strongest activity. Xanthones were found in propolis for the first time; *Garcinia mangostana* (Mangosteen) was the most probable plant source. In addition, this is the first report on the chemical composition and bioactivity of propolis from *T*. *melanoleuca*.

## Introduction

Propolis is a resinous material collected by bees from various plant exudates. Bees use propolis to narrow the nest entrances, seal cracks and embalm dead organisms inside the hive. The antibiotic properties of propolis provide a healthy hive environment for the honeybee colony. Propolis is an apicultural product that has been used for its biological properties, as an alternative medicine and for disease prevention, in different parts of the world. The chemical composition of propolis depends on the collection site, available plant sources and bee species [1], [2]. Several species of bees produce propolis, including *Apis mellifera* and stingless bees (Meliponini) [3], [4].

Stingless bees are widespread over tropical and some subtropical regions of the world [5], [6]. They are the major visitors of many flowering plants in the tropics. Propolis from stingless bees is well known for its therapeutic properties, including antimicrobial, antitumor and antioxidant activities [7], [8]. In Thailand, *Tetragonula laeviceps* is widely distributed and important because it is kept by local population and produces a large amount of propolis [9].

Research on the composition and biological activities of native Thai stingless bee propolis is scarce, although information on its chemical composition and bioactive compounds would be highly beneficial. This study investigated the chemical composition and antimicrobial activity of propolis of two native Thai stingless bee species, *Tetragonula laeviceps* and *Tetrigona melanoleuca*. Here we report, for the first time, information about *T*. *melanoleuca* propolis.

## Materials and Methods

### Ethics Statement

No specific permits were required for the described field studies. All field work was conducted on private land and with owner permission. The field studies did not involve endangered or protected species.

### Propolis samples

Three *Tetragonula laeviceps* propolis samples were collected from Trat Province in eastern Thailand (12° 21′ N, 102° 25′ E) in December 2009. The *Tetrigona melanoleuca* propolis sample was collected from Chiang Mai Province in northern Thailand (18° 48′ N, 98° 57′ E) in February 2012. The propolis samples were collected from honeypots and scraping from the nests.

### GC/MS analysis

Propolis samples (three of *Tetragonula laeviceps* and one of *Tetrigona melanoleuca*) were extracted with 70% ethanol (1:10, w/v) at room temperature for 24 h (3 times). (see supplement S1 Fig). The propolis extracts were evaporated to dryness and silylated using N,O-Bis(trimethylsilyl)trifluoroacetamide (BSTFA). Five milligrams of dry ethanol extract were mixed with 50 μl of dry pyridine and 75 μl of BSTFA, heated at 80°C for 20 min and analyzed by GC/MS. The GC/MS analysis was performed with a Hewlett Packard Gas Chromatograph 5890 Series II Plus linked to a Hewlett Packard 5972 mass spectrometer system equipped with a 23 m long, 0.25 mm id and 0.5 μm film thickness HP5-MS capillary column. The temperature was programmed from 100 to 310°C at a rate of 5°C/min. Helium was used as the carrier gas with a flow rate 0.7 ml/min, split ratio of 1:80, injector temperature of 280°C and ionization voltage of 70 eV.

### Extraction and isolation

NMR spectra: ^1^H NMR (600 MHz) and ^13^C NMR (150 MHz), Bruker AV 600. The NMR solvents are indicated in the Supplementary files together with the corresponding MNR spectra.

#### *Tetragonula laeviceps* propolis

*Tetragonula laeviceps* propolis (200 g) was extracted with 70% ethanol (1:10, w/v) at room temperature for 24 h (3 times) (see supplement S1 Fig). The ethanol extract was concentrated under vacuum until has a volume of 3 L (approximately) and extracted successively with petroleum ether (3 times) and ethyl acetate (3 times). The extracts obtained were evaporated to give 5 g PE and 6.6 g EtOAc dry residue. A part of PE extract (4.5 g) was subjected to column chromatography with silica gel using a PE—EtOAc gradient system to give 22 fractions (A-V). Fraction L (25% PE—EtOAc elute, 40 mg) was subjected to preparative TLC (mobile phase PE—EtOAc 7:4) to obtain α-mangostin **1** (2 mg) [10]. Fractions F and G were combined (186 mg) and subjected to Lobar LiChroprep Si 60 Merck column (40–63 μm) with a PE—EtOAc gradient system to give 26 fractions (F01-F26). Fractions F09, F11 and F14 gave mangostanin **2** (4.9 mg) [10], 8-deoxygartanin **3** (4.6 mg) [11] and gartanin **4** (2.8 mg) [12], respectively. Fraction F21 (10% EtOAc elute, 20 mg) was subjected to preparative TLC (mobile phase PE—EtOAc 9:1, three-fold development) to obtain dipterocarpol **5** (4.7 mg) [13]. Fraction U (10% EtOAc elute, 38 mg) was subjected to preparative TLC (mobile phase CHCl_3_—MeOH 15:1) to obtain γ-mangostin **6** (1.4 mg) [14]. A part of ethyl acetate extract (4.9 g) was extracted with CHCl_3_ (3 times) and evaporated to give 2.5 g dry residue. The CHCl_3_ extract was subjected to silica gel column chromatography with a CHCl_3_—EtOAc gradient system to give 21 fractions (01–21). Fractions 05, 07 and 08 were combined (210 mg) and subjected to Lobar LiChroprep Si 60 Merck column (40–63 μm) with a CHCl_3_—EtOAc gradient system to give 14 fractions (0501–0514). Fraction 0501 gave garcinone B **7** (2.3 mg) [15]. Fraction 0512 (10% EtOAc elute, 13.5 mg) was subjected to preparative TLC (mobile phase CHCl_3_—EtOAc 7:3) to obtain methylpinoresinol **8** (4.7 mg) [16].

#### *Tetrigona melanoleuca* propolis

*Tetrigona melanoleuca* propolis (370 g) was extracted with 70% ethanol (1:10, w/v) at room temperature for 24 h (3 times) (see supplement S1 Fig). The ethanol extract was concentrated under vacuum until has a volume of 3 L (approximately) and extracted successively with petroleum ether (2 times). The petroleum ether extract was evaporated to give 32 g dry residue. A part of PE extract (20 g) was subjected to column chromatography on silica gel with a PE—CH_2_Cl_2_ gradient system to give 21 fractions (A-U). Fraction J, K and L (30% CH_2_Cl_2_ elute, 1.3 g) was re-chromatographed on silica gel with a PE—EtOAc gradient system to give 22 fractions (J1–J22). Fraction J12 (11% EtOAc elute, 20 mg) was subjected to preparative TLC (mobile phase PE—EtOAc 8:2) to obtain a mixture of ursolic and oleanolic aldehydes, **9** and **10** (14.3 mg) [17], [18]. Fraction J13 (190 mg) was subjected to Lobar LiChroprep Si 60 Merck column (40–63 μm) with a PE—EtOAc gradient system to give 11 fractions (J1301-J1311). Fraction J1308 (4% EtOAc elute, 20 mg) was subjected to preparative TLC (mobile phase PE—EtOAc 8:2) to obtain dipterocarpol **5** (12.5 mg) **[** 13 **]**. Fraction T from the PE extract (100% EtOAc elute, 2.2 g) was re-chromatographed on silica gel with a PE—EtOAc gradient system to give 12 fractions (T01-T12). Fraction T05 (194 mg) was subjected to Lobar LiChroprep Si 60 Merck column (40–63 μm) with a PE—EtOAc gradient system to give 22 fractions (T0501-T0522) and fraction T0504 gave 3-*O*-acetyl ursolic acid **11** (5.6 mg) [19]. Fraction T0513 (4% EtOAc elute, 20 mg) was subjected to preparative TLC (mobile phase PE—EtOAc 8:2) to obtain ocotillone I **12** (5.5 mg) [20]. Fraction T0515 (4% EtOAc elute, 20 mg) was subjected to preparative TLC (mobile phase PE—EtOAc 8:2) to obtain ocotillone II **13** (4.8 mg) [21]. Fraction T10 was purified on silica gel column with a CHCl_3_—EtOAc gradient system to give a mixture of cabralealactone and isocabralealactone, **14** and **15** (2.9 mg) [22], [23].

All structures were elucidated using NMR (1D and 2D) spectral data (S2–S14 Figs) and compared with the literature.

### Antibacterial assay

The antibacterial activity of propolis ethanolic extracts and isolated compounds were investigated. The antibacterial assay was determined by dilution method, measuring the minimal inhibitory concentration (MIC) value in a 96-well microtiter plate [24]. Eleven test microorganisms; *Listeria monocytogenes* DMST 17303, *Micrococcus luteus* DMST 15503, *Pseudomonas aeruginosa* ATCC 9027, *Staphylococcus epidermidis* DMST 15505, *Streptococcus pyogenes* DMST 17020, methicillin-resistant *Staphylococcus aureus* (MRSA) DMST 20625, *Serratia marcescens* DMST 21632, *Salmonella typhimurium* DMST 562, *Bacillus cereus* TISTR 687, *Escherichia coli* ATCC 25922 and *S*. *aureus* TISTR 517 were used to test antimicrobial activity. All isolated compounds were dissolved by Dimethyl Sulfoxide (DMSO) for the antimicrobial test. Tested bacteria were cultured in Mueller Hinton broth (MHB) and incubated at 37°C for 24 hours. Bacteria were suspended in MHB by adjusting to 0.5 McFarland, yielding a final density of 10^8^ cfu/ml. The ethanol extracts of propolis were prepared in concentrations ranging from 0.25 mg/ml to 128 mg/ml. In addition, pure compounds were prepared in concentrations ranging from 0.39 μg/ml to 25 μg/ml for this assay. The two fold serial dilutions of propolis extract or isolated compounds (180 μl) and test strain solution (20 μl) were added into each well of the microtiter plate (Cell Culture Plates, metric volume 0.36 ml). Positive (broth and inoculum) and negative (sterile broth) growth controls were used to compare. The MICs were determined as the lowest concentrations of compounds preventing visible bacteria growth. The minimum bactericidal concentrations were determined by subculturing 10 μl of inoculum from the MIC wells onto Mueller Hinton agar plates. The MBCs were determined as the lowest concentration that prevented visible growth of bacteria subcultures on the agar plate. Each sample was tested in triplicate. Gentamicin was used as positive control. The MICs and MBCs of gentamicin ranged from 0.02–0.78 mg/ml and 0.02–1.56 mg/ml, respectively.

### Statistical analysis

Statistical significance was evaluated using one way analysis of variance (ANOVA) by SPSS version 16 (SPSS Inc.).

## Results and Discussion

### Chemical composition

The chemical profiles of propolis ethanol extracts were studied by GC-MS (after silylation). All three samples of *T*. *laeviceps* propolis displayed identical profiles, while *T*. *melanoleuca* propolis was different from them (Total Ion Chromatograms: S15 Fig). Moreover, the GC-MS profiles for propolis of both species did not coincide with any known propolis type and demonstrated the lack of plant secondary metabolites previously found in propolis. For this reason, it was necessary to isolate and identify individual compounds in order to reveal the specific chemistry and, if possible, the plant origin of the studied stingless bee propolis.

**Fig 1:**
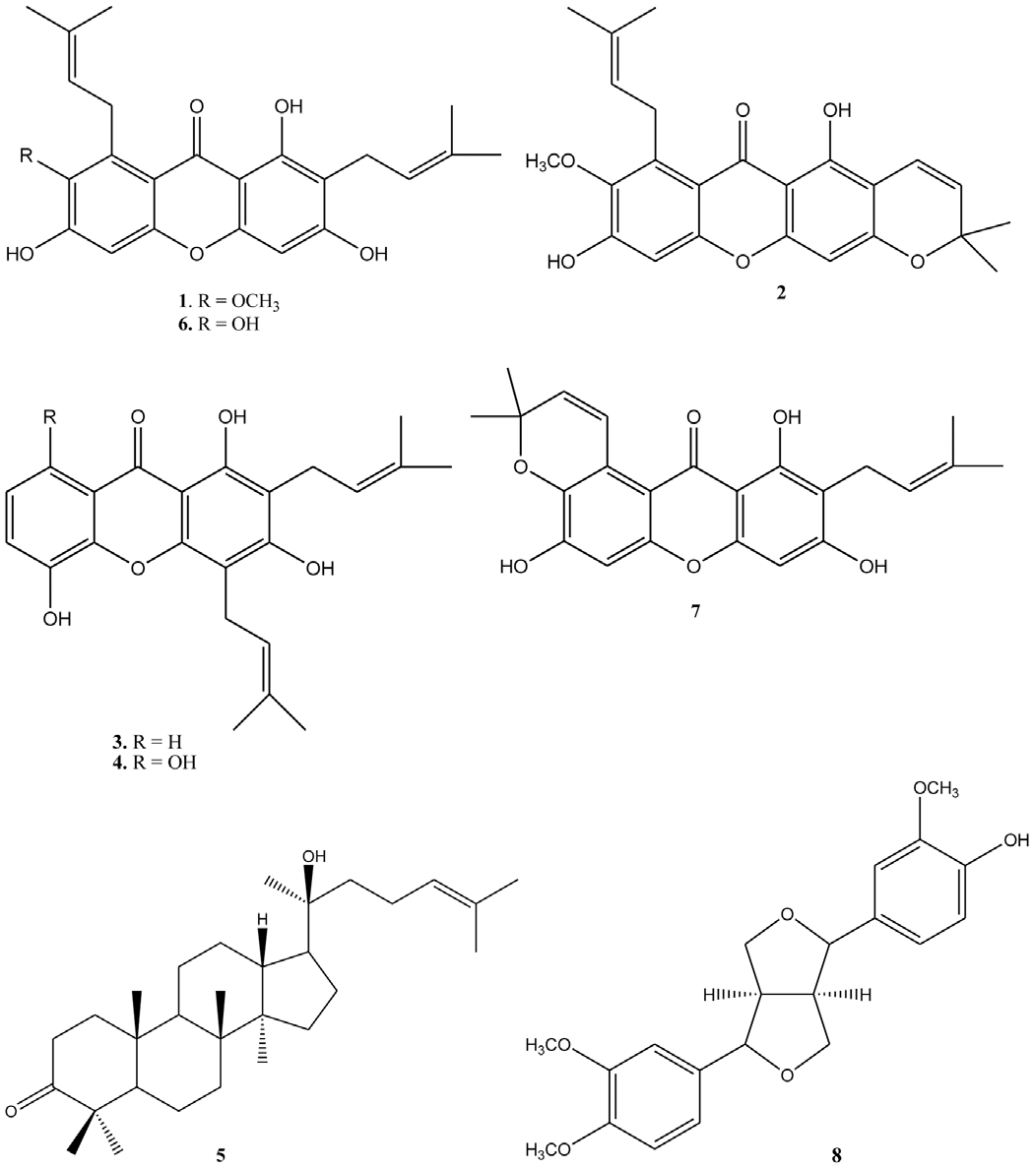
Compounds isolated from of *Tetragonula laeviceps* propolis. α-mangostin 1, mangostanin 2, 8-deoxygartanin 3, gartanin 4, γ-mangostin 6 and garcinone B 7. The dammarane triterpene dipterocarpol 5 and the furofurane lignane methylpinoresinol 8 were isolated from *T*. *laeviceps* propolis.

The petrol ether fraction of the ethanol extract of *T*. *laeviceps* propolis was subjected to repeated chromatographic separation and six individual compounds were isolated and characterized (Fig 1), among which the prenylated xanthones: α-mangostin **1**, mangostanin **2**, 8-deoxygartanin **3**, gartanin **4**, γ-mangostin **6** and the dammarane triterpene dipterocarpol **5**. From the ethyl acetate fraction of the ethanol extract, a further xanthone garcinone B **7** and the furofurane lignane methylpinoresinol **8** were also isolated and identified. It is important to note that the xanthones are new propolis constituents and the first xanthones to be isolated from the propolis. Prenylated xanthones have been recognized as major secondary metabolites of *Garcinia mangostana* (Mangosteen), and all the xanthones (**1**–**4**, **6**, **7**) have been previously isolated from the pericarp and young fruit of mangosteen [10], [25], [26]. As it is well known that bees collect resinous material from the surfaces of young leaves, fruits and buds, *G*. *mangostana* is the most probable plant source of *T*. *laeviceps* propolis. The mangosteen trees are widespread across India, Myanmar, Malaysia, the Philippines, Sri Lanka and Thailand. The pericarp has been used in Thai indigenous medicine for the treatment of trauma, diarrhea and skin infections for a long time [27], [28]. Previous studies have demonstrated antibacterial activity of xanthones and extracts obtained from Mangosteen [29].

**Fig 2:**
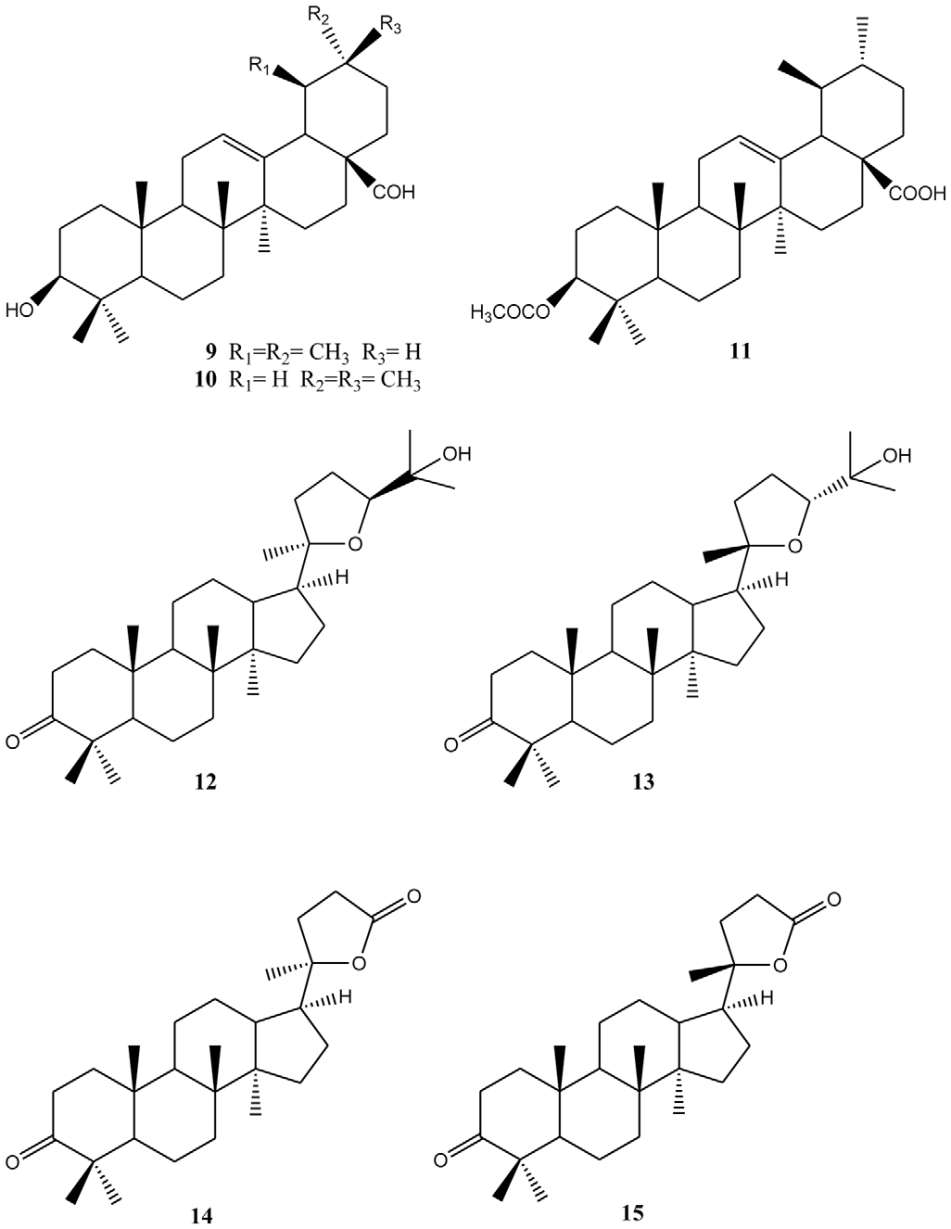
Compounds isolated from of *Tetrigona melanoleuca* propolis. 3-*O*-acetyl ursolic acid 11, dipterocarpol 5, ocotillone I 12, ocotillone II 13, and mixtures of ursolic and oleanolic aldehydes 9–10, and cabralealactones 14–15 were isolated from *T*. *melanoleuca* propolis.

From petrol ether fraction of the ethanol extract of *T*. *melanoleuca* propolis, the triterpenes 3-*O*-acetyl ursolic acid **11**, dipterocarpol **5**, ocotillone I **12**, ocotillone II **13**, and two mixtures: of ursolic and oleanolic aldehydes **9**–**10**, and of cabralealactones **14**–**15**, were isolated after repeated chromatographic procedures. Their structures were confirmed by comparison of their NMR spectra with literature data. (S10–S14 Figs) All of these triterpenes are new propolis constituents (Fig 2). Their presence in this propolis provides valuable chemotaxonomic information about the plants from which the stingless bees *T*. *melanoleuca* collected resin. The simultaneous occurrence of dammarane (**5**, **12**–**15**), ursane and oleanane derivatives (**9**, **10**) has been described as an indicator of the presence of dammar in the mixture [30]. Dammar is a triterpenic resin produced by trees belonging to the family Dipterocarpaceae. Dammar was reported to possess antiviral activities and to be protective against *in vitro* low density lipoprotein (LDL) oxidation [31].

A further confirmation of origin of *T*. *melanoleuca* propolis from dammar resin was the identification in its GC-MS profile of other known dammar components: 2,3-dihydroxyolean-12-en-28-oic (maslinic) acid and 2,3-dihydroxyurs-12-en-28-oic (corosolic) acid were identified by comparison of the spectra of their silylated derivatives (S16 Fig) with literature data [32]. Two other acids were tentatively identified as 2,3-dihydroxyoleanadien-28-oic acid and 2,3-dihydroxyursadien-28-oic acid, based on comparison of the mass spectra of their TMS derivatives (S17 Fig) with the mass spectra of underivatized 2,3-dihydroxyoleanadien-28-oic acid and 2,3-dihydroxyursadien-28-oic acid [30], mass spectra of 2,3-diacetyloxyoleanadien-28-oic acid and 2,3-diacetyloxyursadien-28-oic acid [33] and mass spectra of silylated maslinic and corosolic acids [30]. The major peak in the TIC chromatogram (23% of TIC) belonged to 2,3-dihydroxyursadien-28-oic acid and this is characteristic for the specific chemical profile of Dipterocarpaceae resins, which has been previously demonstrated by Burger et al. [30]. Actually, different stingless bee species are known to collect resin from dipterocarp trees [34]; stingless bees are even called “dammar bees” in some parts of India [35]. Nonetheless, the reported triterpenes (**5, 9–15**) have not previously been found in stingless bee propolis.

### Antimicrobial activity of extracts and isolated compounds

The antimicrobial activity of ethanol extract of *T*. *laeviceps* propolis and *T*. *melanoleuca* propolis samples was investigated. Eleven bacteria strains were used to test the minimal inhibitory concentration (MIC) and the minimum bactericidal concentration (MBC). The ethanol extract of *T*. *laeviceps* propolis displayed mild antimicrobial activity against *S*. *epidermidis* (MIC = 0.13 mg/ml; MBC = 32 mg/ml). The MICs and MBCs of *T*. *laeviceps* propolis ranged from 0.13–16 mg/ml and 1–128 mg/ml, respectively. The results for the total extract of *T*. *laeviceps* propolis against *S*. *aureus* (MIC = 1 mg/ml; MBC = 16 mg/ml) are of the same order of magnitude as the published by Kaewmuangmoon et al. (2012) [36]. The ethanol extract of *T*. *melanoleuca* propolis suppressed the development of *S*. *aureus*, methicillin-resistant *Staphylococcus aureus* and *E*. *coli*. The MICs and MBCs ranged from 2–16 mg/ml and 16–128 mg/ml, respectively. In general, the MIC of the total extracts were close to or above the value of 1 mg/ml, accepted as the highest relevant value in studies of the antibacterial activity of natural product extracts [37]

The results demonstrated that, of all tested organisms, *S*. *epidermidis* was the most sensitive and *S*. *marcescens* the least sensitive (MIC = 16 mg/ml; MBC = 128 mg/ml). As can be seen, propolis displayed both bacteriostatic and bactericidal actions depending on the concentration, type of propolis, type of bacteria tested and methodologies to determine antimicrobial activity [38]. The ethanol extract of propolis from *T*. *melanoleuca* showed less activity against tested microorganism compared with *T*. *laeviceps*.

Furthermore, in search of the active principles, isolated pure compounds from both propolis types were tested for their antibacterial activity against several bacteria. The constituents of *T*. *laeviceps* propolis showed good activity (Table 1), especially against *S*. *pyogenes* (MIC = 0.78–25 μg/ml; MBC = 1.30–25 μg/ml), followed by *L*. *monocytogenes* (MIC = 0.78–25 μg/ml; MBC = >25 μg/ml). Concerning statistical analysis results, α-mangostin **1** was the most important antibacterial compound among the eight active compounds identified in the *T*. *laeviceps* propolis samples (*p* < 0.05). It is well known that the mangostins **1** and **6** are the major bioactive compounds in the mangosteen [39]. The antibacterial activities of *T*. *laeviceps* propolis extract could be attributed to the xanthones, especially **1** and **6**.

**Table 1:** Antimicrobial activities of isolated compound from *Tetragonula laeviceps* propolis.

Compound (μg/ml)	Gram-positive bacteria							Gram-negative bacteria			
	*B*.*c*.	*L*.*m*.	*M*.*l*.	*S*.*a*.	*S*.*e*.	*S*.*p*.	MRSA	*E*.*c*.	*P*.*a*.	*S*.*t*.	*S*.*m*.
**MIC**											
α-mangostin^a^ **1**	3.13	0.78	6.25	3.13	1.56	0.78	3.13	12.5	12.5	12.5	12.5
Mangostanin^c^ **2**	12.5	0.78	25	12.5	25	3.13	12.5	25	25	25	25
8-deoxygartanin^b^ **3**	3.13	1.56	25	1.56	1.56	1.56	1.56	25	25	25	25
Gartanin^c^ **4**	25	12.5	25	12.5	25	6.25	12.5	25	25	25	25
Dipterocarpol^d^ ^e^ **5**	25	25	25	>25	>25	6.25	25	25	25	25	25
γ-mangostin^c^ ^d^ **6**	25	12.5	25	25	25	6.25	12.5	25	25	25	25
Garcinone B^b^ **7**	3.13	6.25	3.13	6.25	3.13	1.56	6.25	25	25	25	25
Methylpinoresinol^e^ **8**	25	25	25	25	>25	25	25	25	25	25	25
**MBC**											
α-mangostin^a^ **1**	3.13	>25	8.33	25	25	1.30	>25	>25	25	>25	25
Mangostanin^b^ ^c^ **2**	25	>25	25	>25	>25	10.42	>25	>25	>25	>25	>25
8-deoxygartanin^c^ ^d^ **3**	3.13	>25	>25	>25	>25	25	>25	>25	>25	>25	>25
Gartanin^d^ **4**	>25	>25	>25	>25	>25	12.5	>25	>25	>25	>25	>25
Dipterocarpol^d^ **5**	>25	>25	>25	>25	>25	12.5	>25	>25	>25	>25	>25
γ-mangostin^d^ **6**	25	>25	>25	>25	>25	25	>25	>25	>25	>25	>25
Garcinone B^b^ **7**	3.13	>25	6.25	>25	>25	2.08	>25	>25	>25	>25	>25
Methylpinoresinol^d^ **8**	>25	>25	>25	>25	>25	25	>25	>25	>25	>25	>25

Minimal Inhibitory Concentration (MIC) and Minimum Bactericidal Concentration (MBC) of purified compounds from *Tetragonula laeviceps* against pathogenic bacteria. *B*.*c*.; *Bacillus cereus*, *L*.*m*.; *Listeria monocytogenes*, *M*.*l*.; *Micrococcus luteus*, *S*.*a*.; *Staphylococcus aureus*, *S*.*e*.; *Staphylococcus epidermidis*, *S*.*p*.; *Streptococcus pyogenes*, MRSA; methicillin-resistant *Staphylococcus aureus*, *E*.*c*.; *Escherichia coli*, *P*.*a*.; *Pseudomonas aeruginosa*, *S*.*t*.; *Salmonella typhimurium*, *S*.*m*.; *Serratia marcescens*.

^a,b,c,d,e^ Means with different letters are significant differences for purified compounds.

The triterpenes isolated from *T*. *melanoleuca* propolis exhibited MIC 25 μg/mL against both Gram-positive and Gramm-negative bacteria. The only exception was the mixture of oleanolic and ursolic aldehides (**9** and **10)** with MIC 6.35 μg/mL against *S*. *aureus*. Previous studies have reported the antibacterial activity of these two compounds [40], [41]. The MBC were over 25 μg/mL in all cases, only 25 μg/mL for ocotillone I, ocotillone II and the mixture of cabralealactones against *S*. *aureus*.

## Conclusions

The results of our study have revealed new data about the chemical composition and plant origin of stingless bee propolis from Thailand. They indicate for the first time the plant source, based on taxonomic markers, of the *T*. *laeviceps* propolis in Trat Province: the mangosteen *Garcinia mangostana*. They also indicated for the first time, based on our chemical study of *T*. *melanoleuca* propolis, that these stingless bees collect resin from dipterocarp trees. The antibacterial tests demonstrated some potential of the propolis extract from *T*. *laeviceps* against *S*. *epidermidis*, confirming its use in traditional medicine. The antibacterial activity of individual constituents of the studied propolis has also been proved.

## Supporting Information

S1 Fig Flow chart of extraction and isolation. (DOC)

S2 Fig ^1^H, ^13^C, DEPT, HSQC and HMBC NMR spectra of α-mangostin 1 in acetone-d_6_. (DOC)

S3 Fig ^1^H-NMR spectrum of mangostanin 2 in CDCL_3_. (DOC)

S4 Fig ^1^H, ^13^C and DEPT NMR spectra of 8-deoxygartanin 3 in CDCL_3_. (DOC)

S5 Fig ^1^H, ^13^C and DEPT NMR spectra of gartanin 4 in CDCL_3_. (DOC)

S6 Fig ^1^H, ^13^C and DEPT NMR spectra of dipterocarpol 5 in CDCL_3_. (DOC)

S7 Fig ^1^H-NMR spectrum of γ-mangostin 6 in acetone-d_6_. (DOC)

S8 Fig ^1^H, ^13^C, DEPT, HSQC and HMBC NMR spectra of garcinone B 7 in acetone-d_6_. (DOC)

S9 Fig ^1^H, ^13^C, DEPT, HSQC and HMBC NMR spectra of methylpinoresinol 8 in CDCL_3_. (DOC)

S10 Fig ^1^H-NMR spectrum of mixtures of ursolic and oleanolic aldehydes 9 and 10 in CDCL_3_. (DOC)

S11 Fig ^1^H, ^13^C, DEPT, ^1^H-^1^H COSY, HSQC and HMBC NMR spectra of 3-*O*-acetyl ursolic acid 11 in CDCL_3_. (DOC)

S12 Fig ^1^H, ^13^C and DEPT NMR spectra of ocotillone I 12 in CD_3_OD:CDCL_3_ 2:1. (DOC)

S13 Fig ^1^H, ^13^C, DEPT, ^1^H-^1^H COSY, HSQC and HMBC NMR spectra of ocotillone II 13 in CD_3_OD:CDCL_3_ 2:1. (DOC)

S14 Fig ^1^H, ^13^C and DEPT NMR spectra of mixture of cabralealactones 14–15 in CD_3_OD:CDCL_3_ 2:1. (DOC)

S15 Fig Total ion chromatograms of silylated propolis ethanol extracts. (DOC)

S16 Fig Mass spectra of silylated maslinic and corosolic acids (from GC-MS of sample *T*. *melanoleuca*). (DOC)

S17 Fig Mass spectra of silylated 2,3-dihydroxyoleanadien-28-oic and 2,3-dihydroxyursadien-28-oic acids (from GC-MS of sample *T*. *melanoleuca*). (DOC)
