# Supplementary material for: Antibacterial Compounds from Propolis of Tetragonula laeviceps and Tetrigona melanoleuca (Hymenoptera: Apidae) from Thailand
Source: PLoS One. 2015 May 18;10(5):e0126886. doi: 10.1371/journal.pone.0126886 (PMC4436274; doi:10.1371/journal.pone.0126886)
Supplement: S1 Fig — (DOC) [file pone.0126886.s001.doc]

**S1 Fig. Flow chart of extraction and isolation**

Raw propolis

70 % Ethanol extract

BSTFA

GC-MS

Antibacterial tests

1. Concentration under vacuum

2. Liquid-liquid fractionation

Petroleum ether

Ethyl acetate

Individual compounds

Individual compounds

Column chromatography, preparative TLC

**1, 2, 3, 4, 5, 6** from *Tetragonula laeviceps*

**5, 9** and **10, 11, 12, 13, 14** and **15** from *Tetrigona melanoleuca*

**7, 8** from *Tetragonula laeviceps*

Column chromatography, .ontractedoleum ether PLOS ONE.

FC of yeast of purepreparative TLC
