# Supplementary figures and images for: Antibacterial Compounds from Propolis of Tetragonula laeviceps and Tetrigona melanoleuca (Hymenoptera: Apidae) from Thailand
Source: PLoS One. 2015 May 18;10(5):e0126886. doi: 10.1371/journal.pone.0126886 (PMC4436274; doi:10.1371/journal.pone.0126886)

**S2 Fig. 1H, 13C, DEPT, HSQC and HMBC NMR spectra of α-mangostin 1 in acetone-d6.**


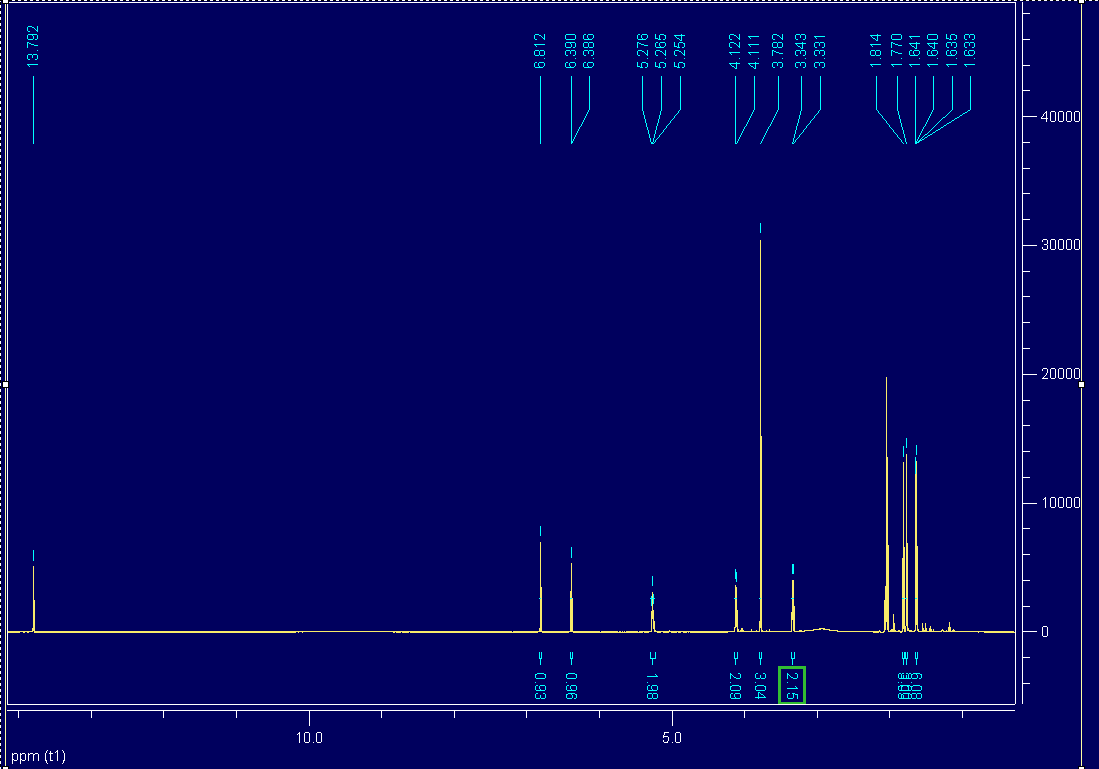


**
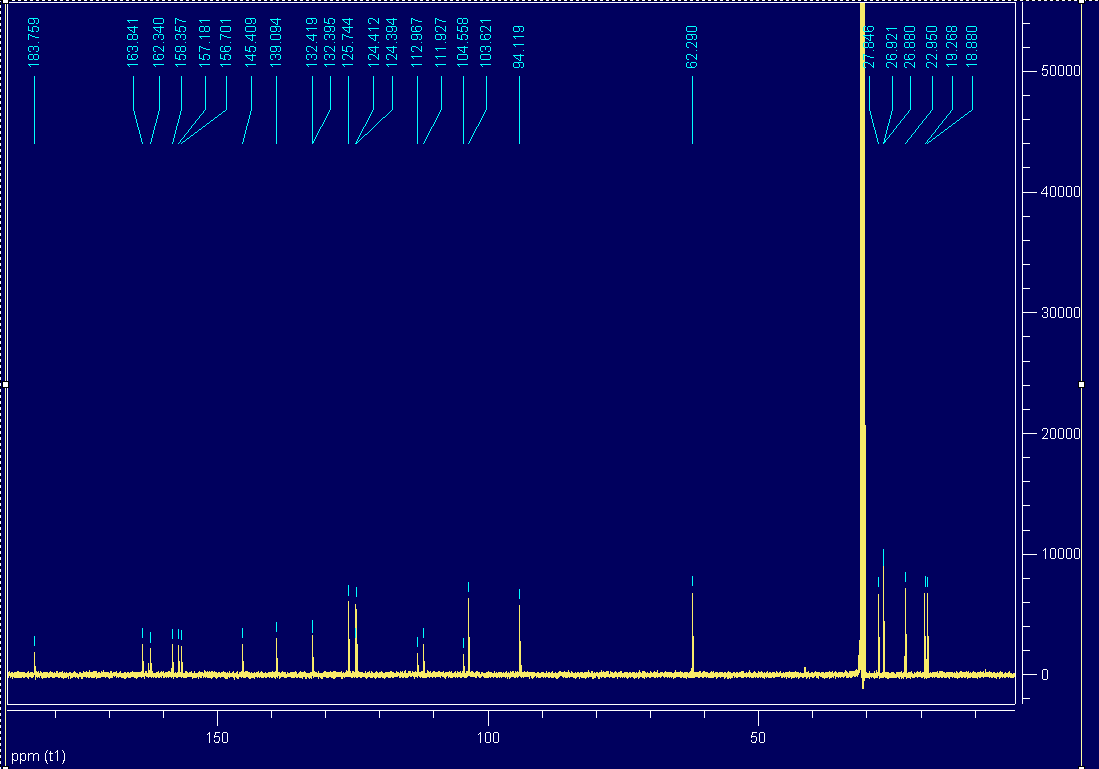
**


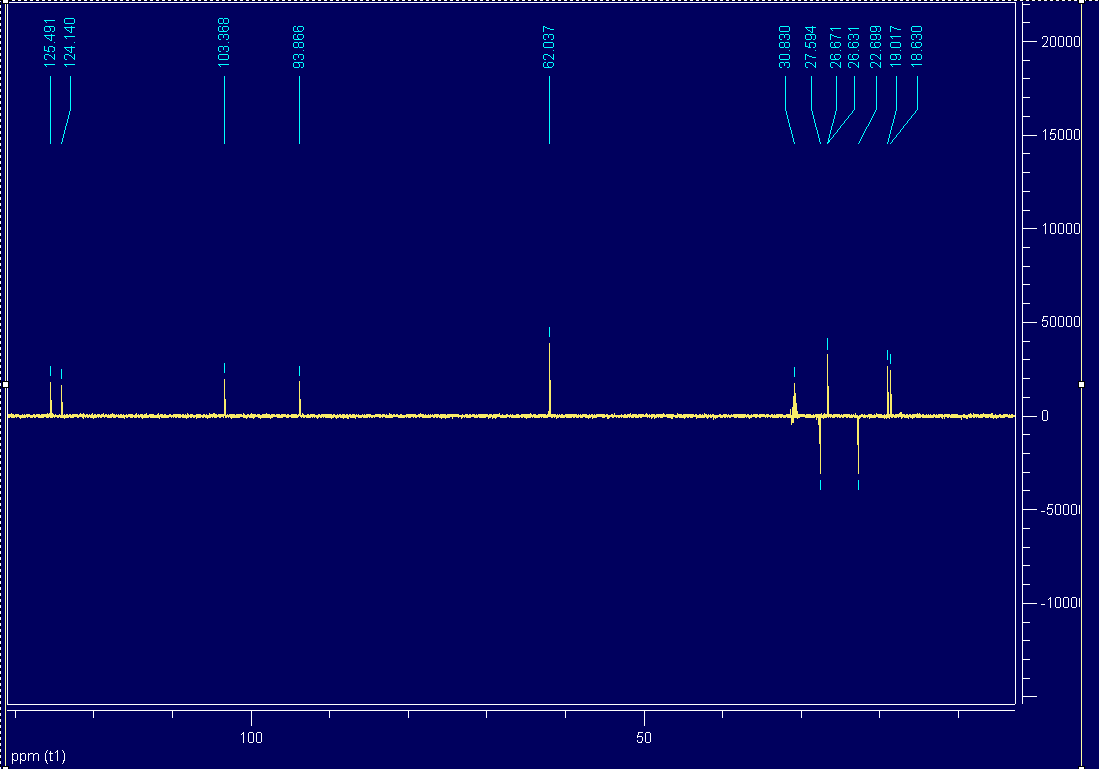


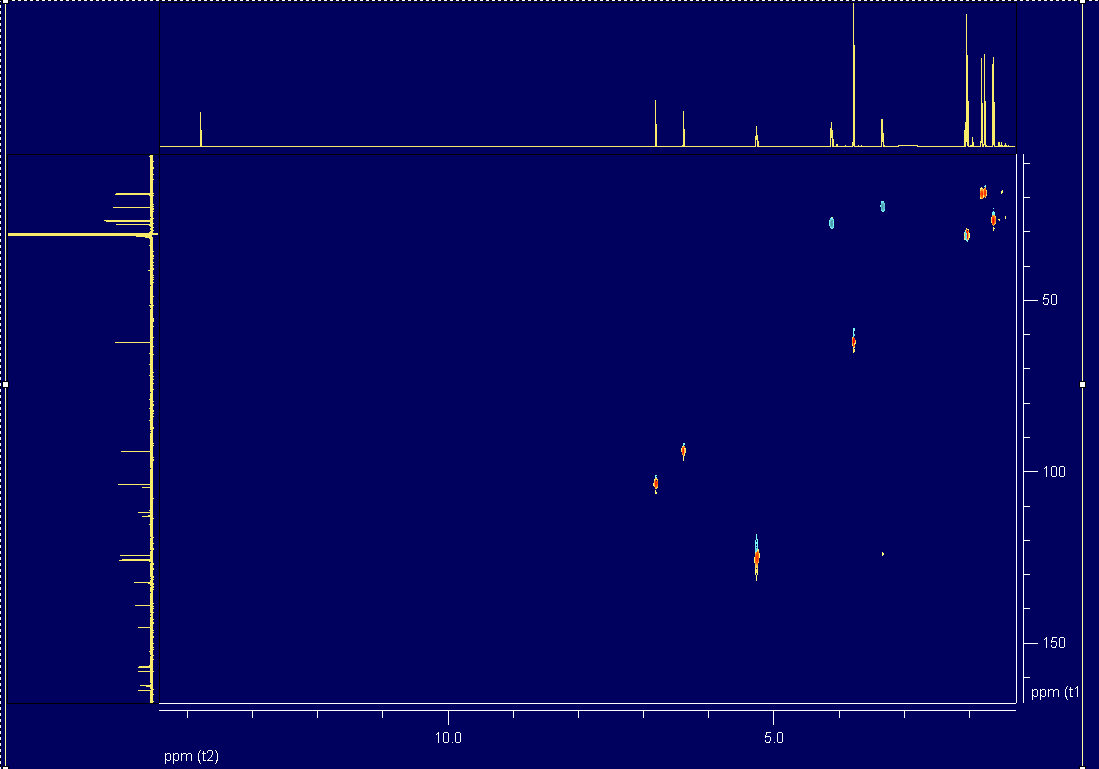


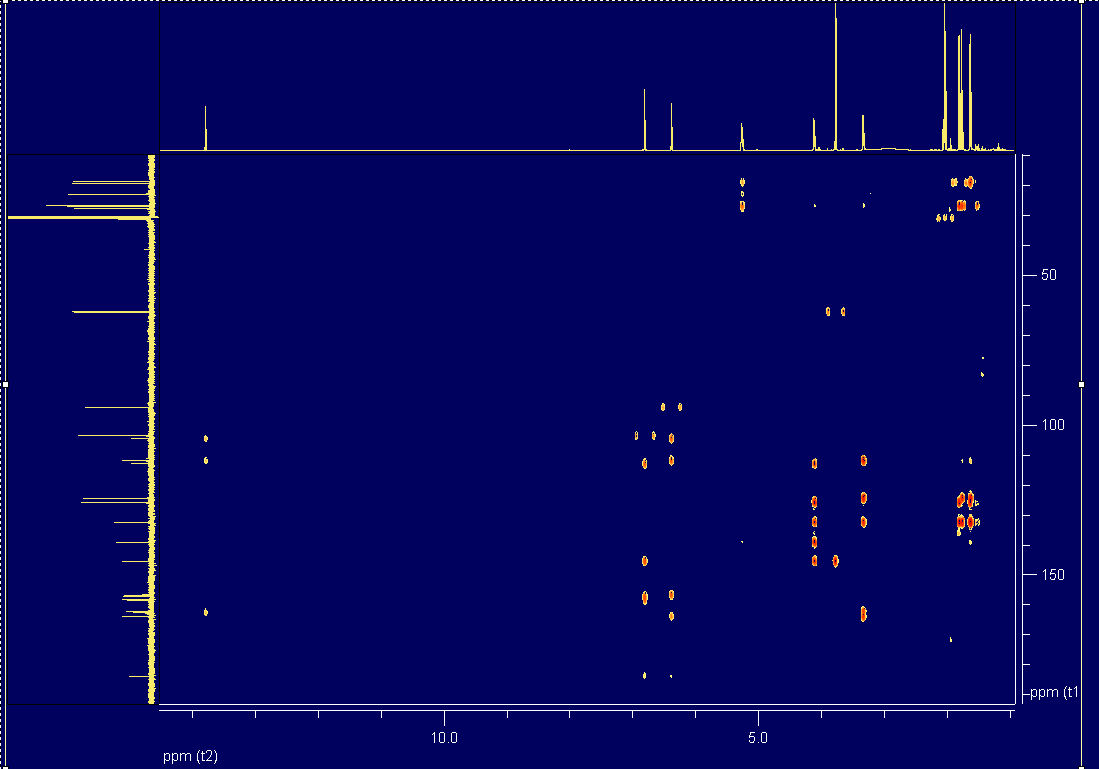

Supplement: S2 Fig — (DOC) [file pone.0126886.s002.doc]

**S3 Fig. 1H-NMR spectrum of mangostanin 2 in CDCL3.**


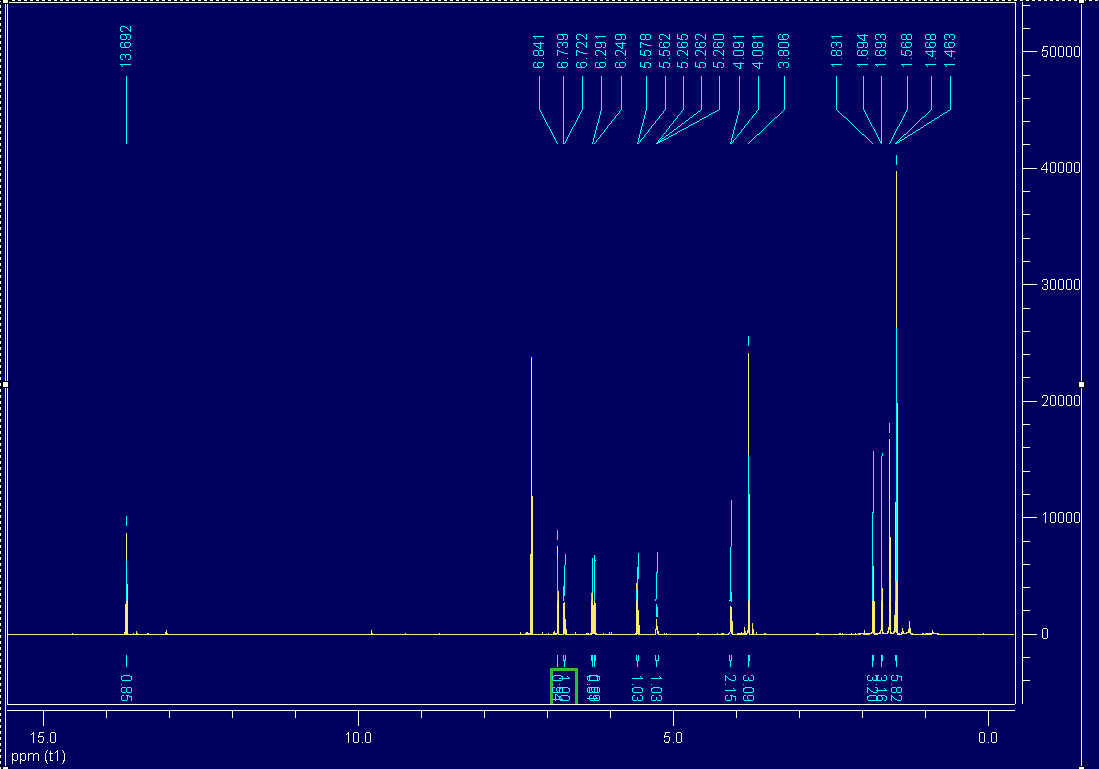

Supplement: S3 Fig — (DOC) [file pone.0126886.s003.doc]

**S4 Fig. 1H, 13C and DEPT NMR spectra of 8-deoxygartanin 3 in CDCL3.**


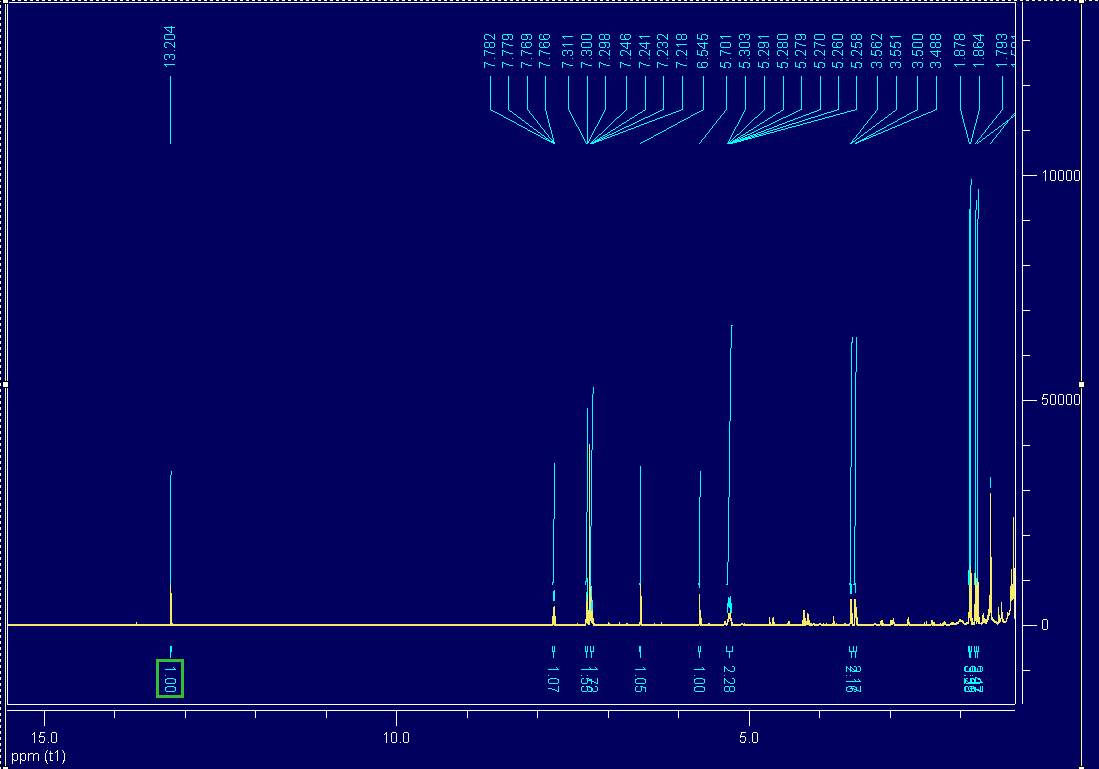


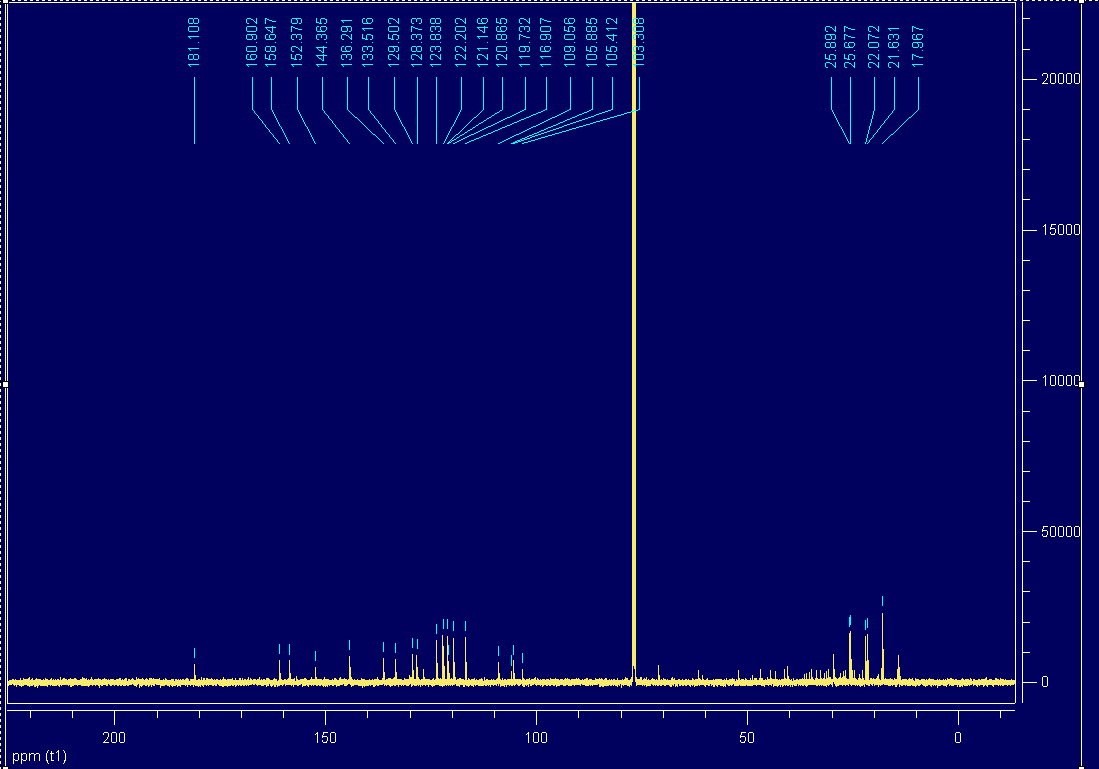


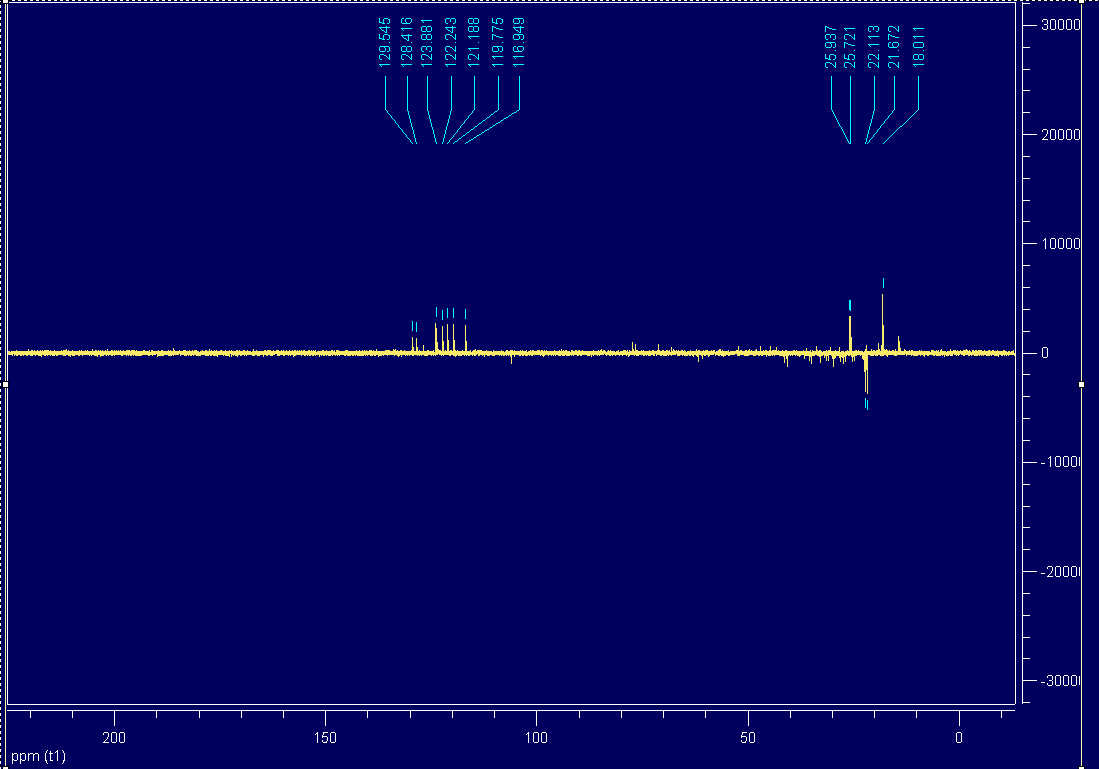

Supplement: S4 Fig — (DOC) [file pone.0126886.s004.doc]

**S5 Fig. 1H, 13C and DEPT NMR spectra of gartanin 4 in CDCL3.**


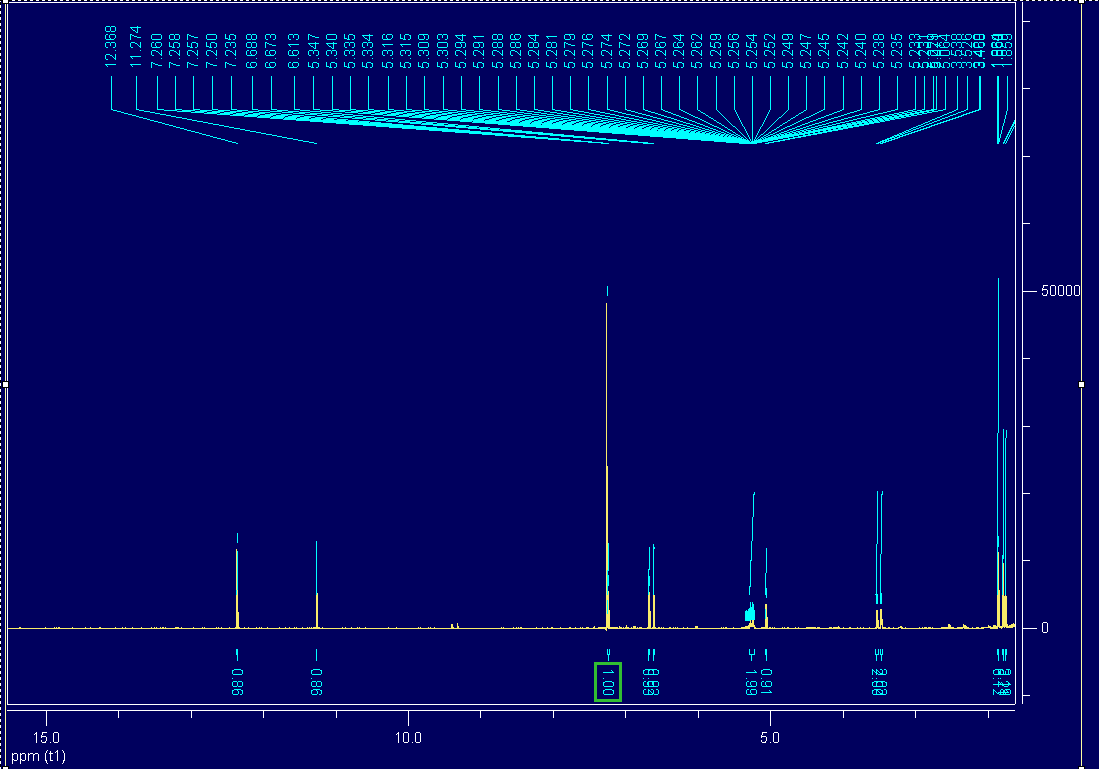


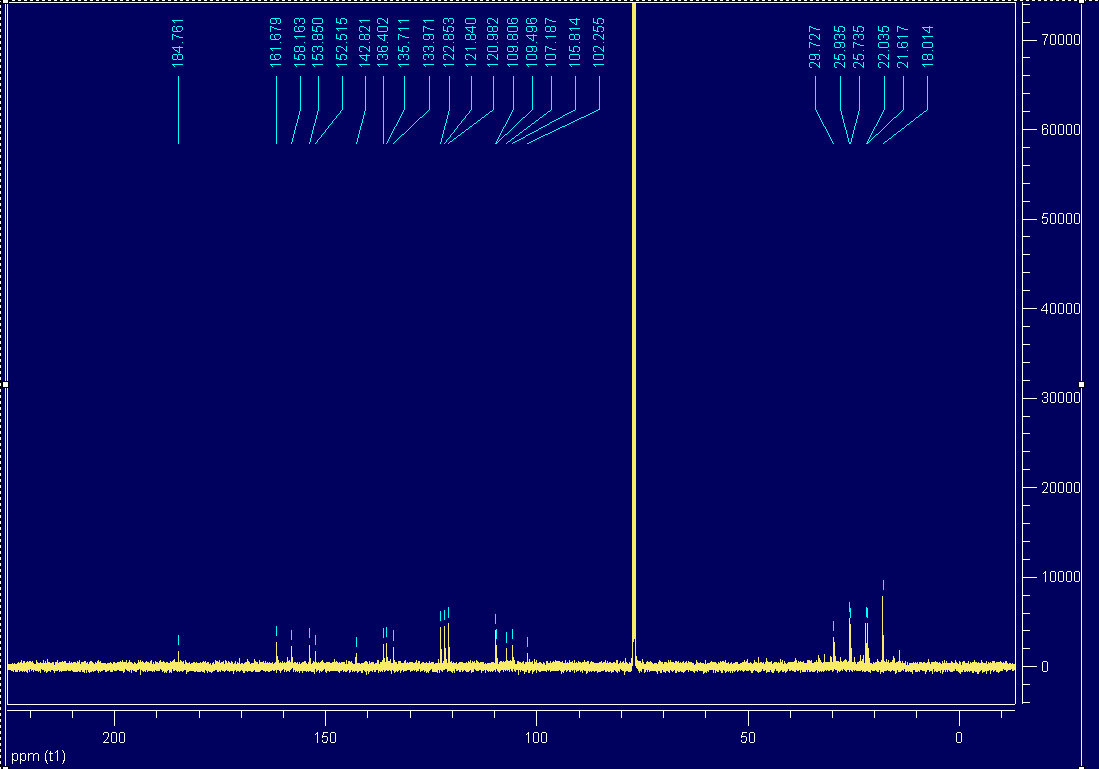


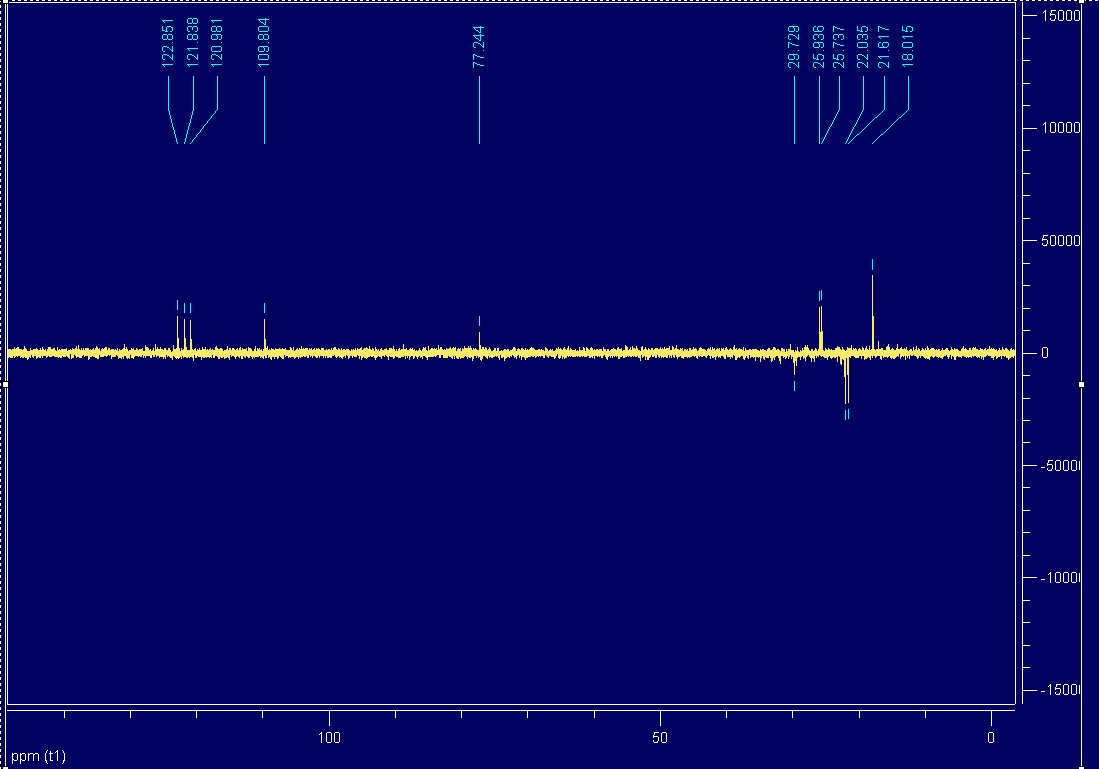

Supplement: S5 Fig — (DOC) [file pone.0126886.s005.doc]

**S6 Fig. 1H, 13C and DEPT NMR spectra of dipterocarpol 5 in CDCL3.**


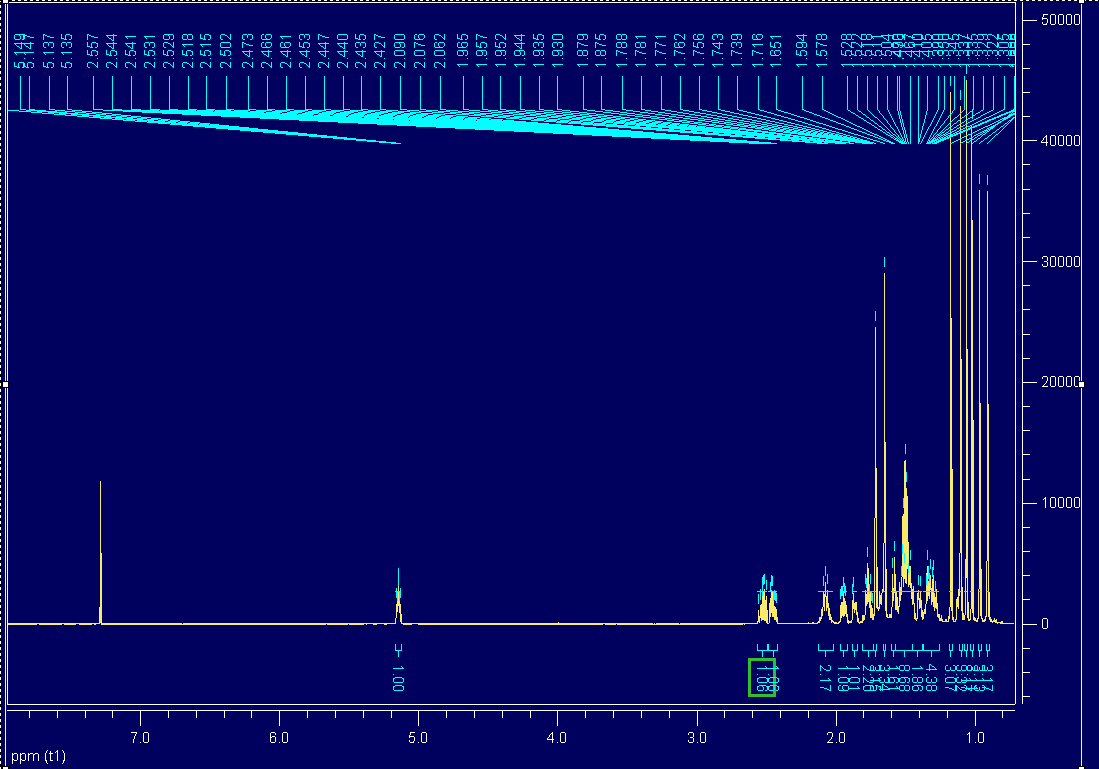


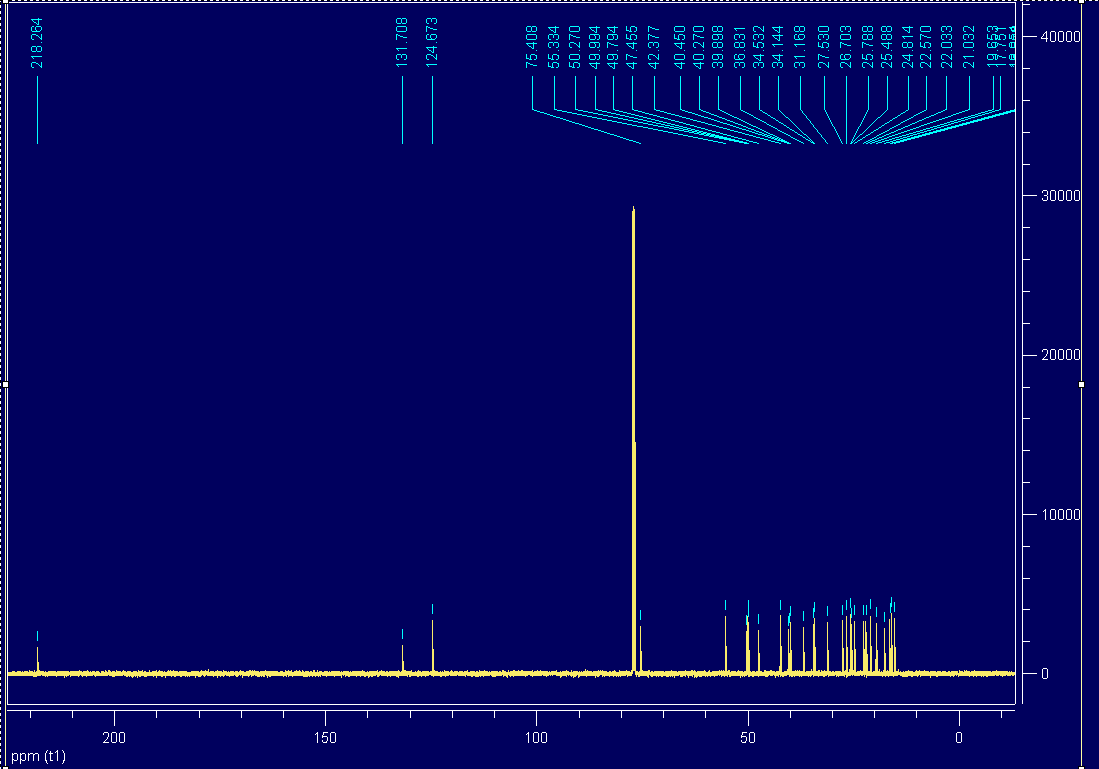


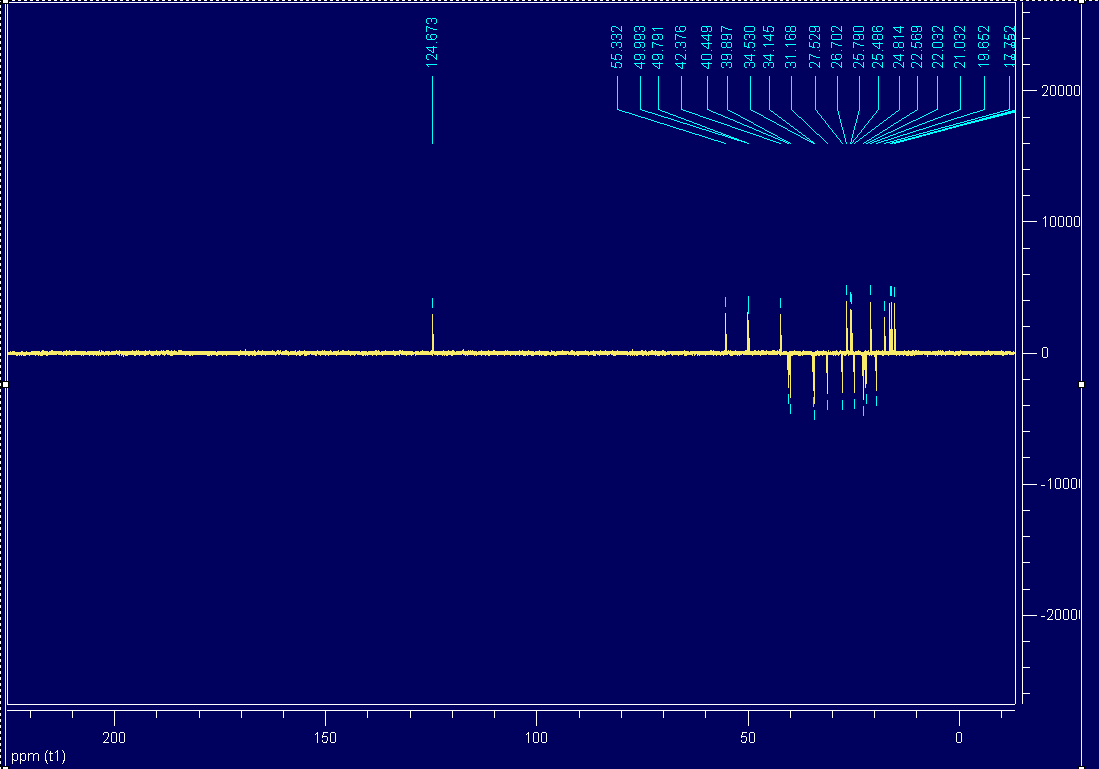

Supplement: S6 Fig — (DOC) [file pone.0126886.s006.doc]

**S7 Fig. 1H-NMR spectrum of γ-mangostin 6 in acetone-d6.**


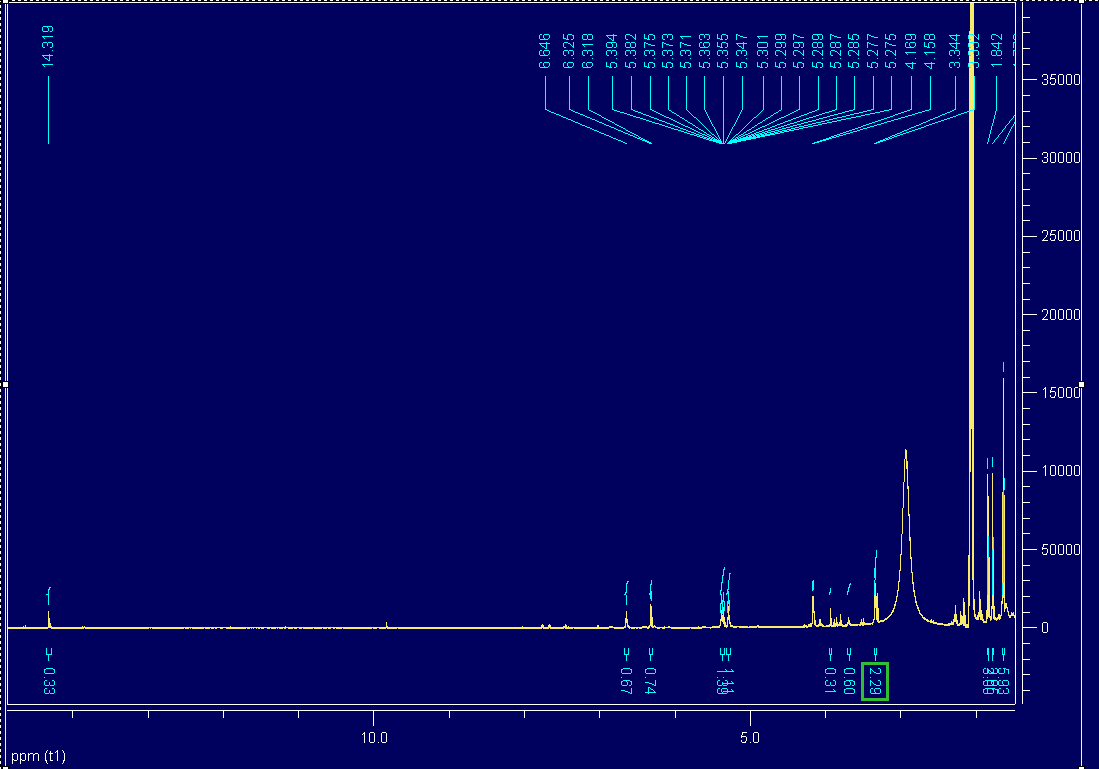

Supplement: S7 Fig — (DOC) [file pone.0126886.s007.doc]

**S8 Fig. 1H, 13C, DEPT, HSQC and HMBC NMR spectra of garcinone B 7 in acetone-d6.**


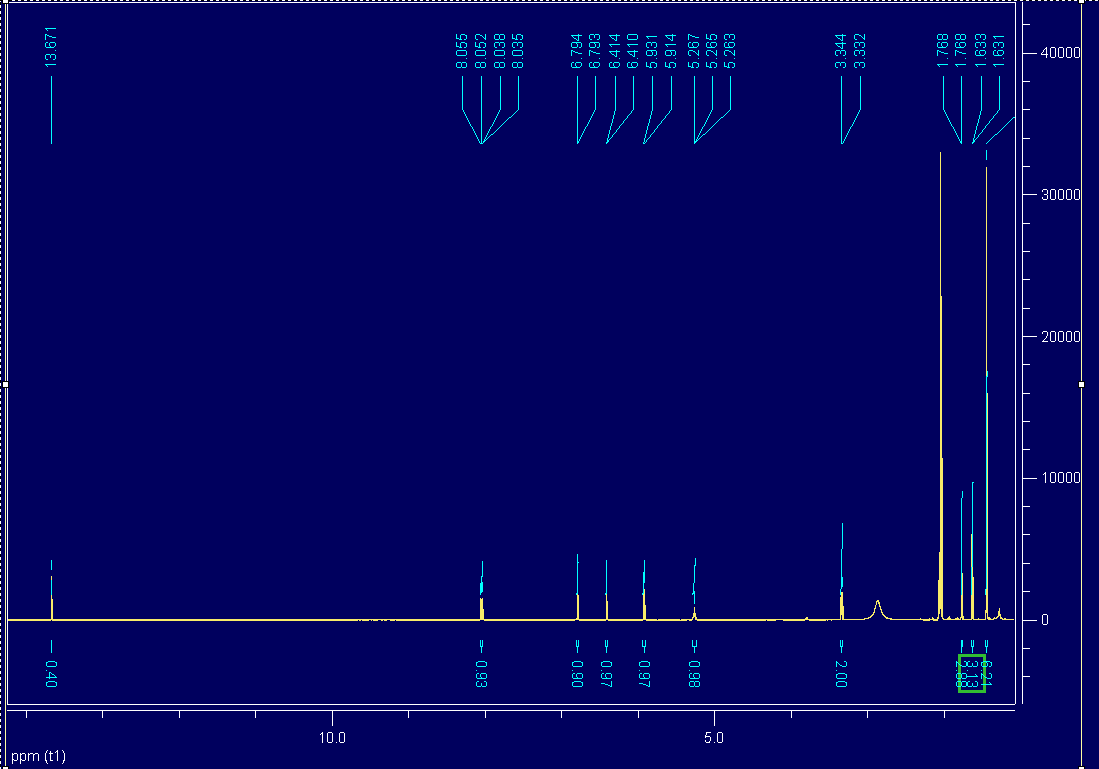


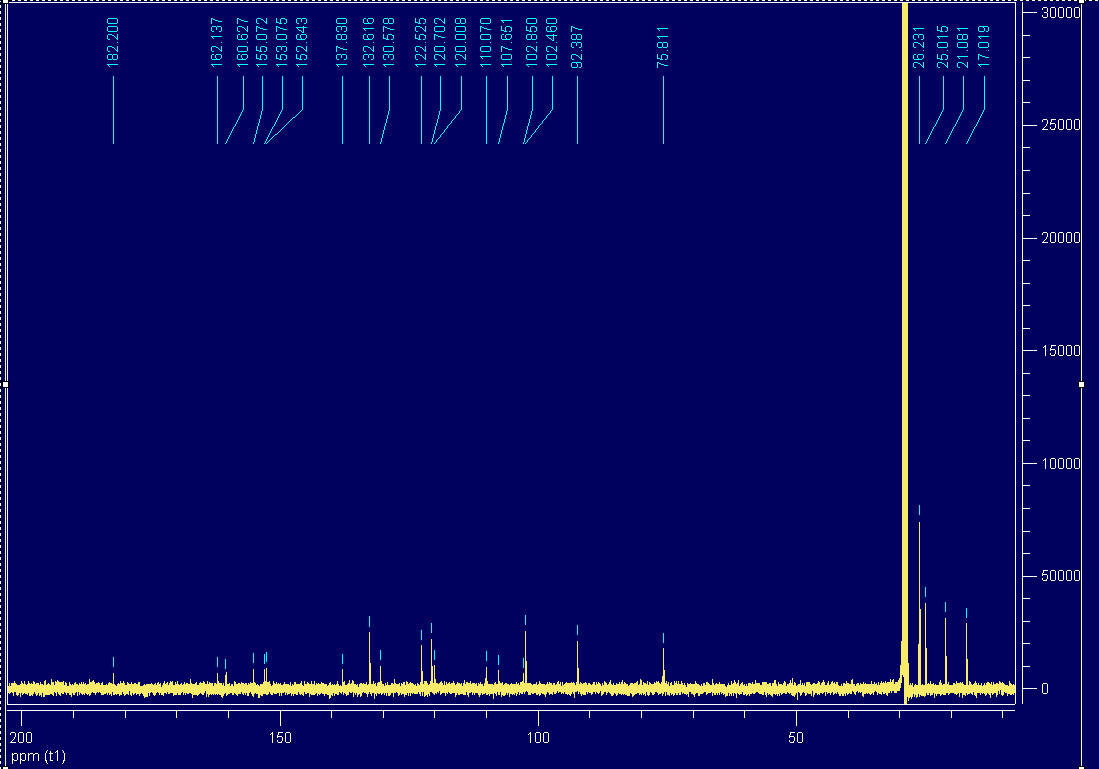


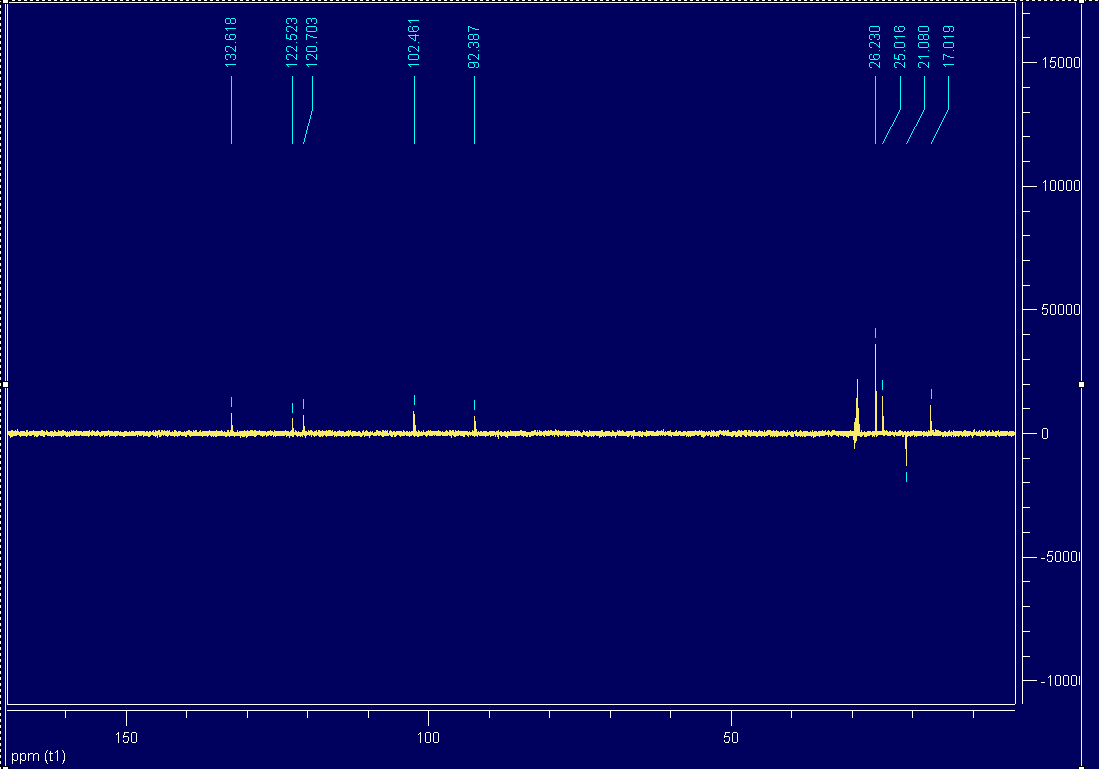


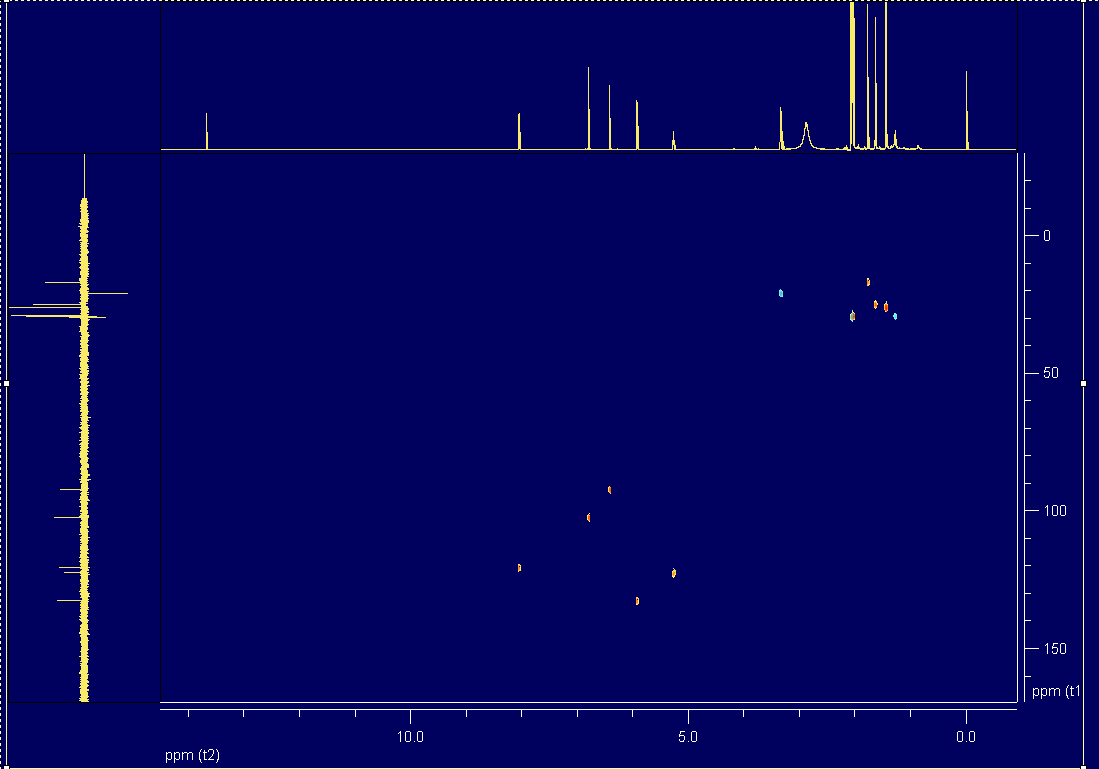


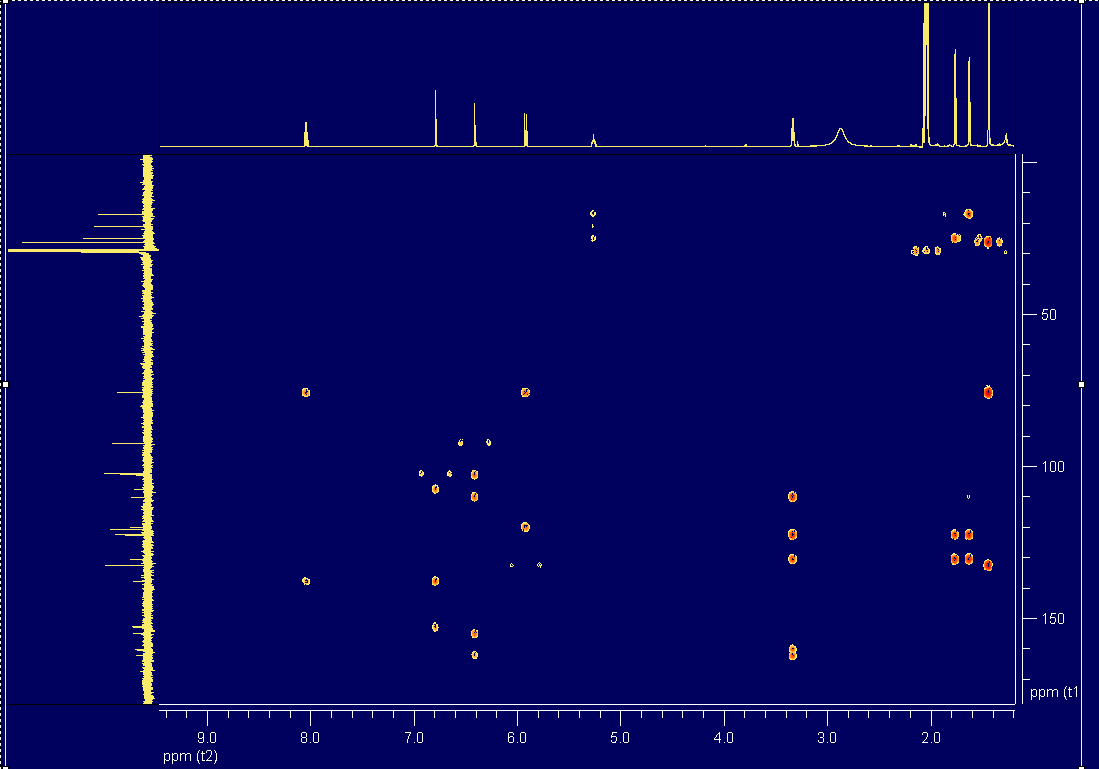

Supplement: S8 Fig — (DOC) [file pone.0126886.s008.doc]

**S9 Fig. 1H, 13C, DEPT, HSQC and HMBC NMR spectra of methylpinoresinol 8 in CDCL3.**


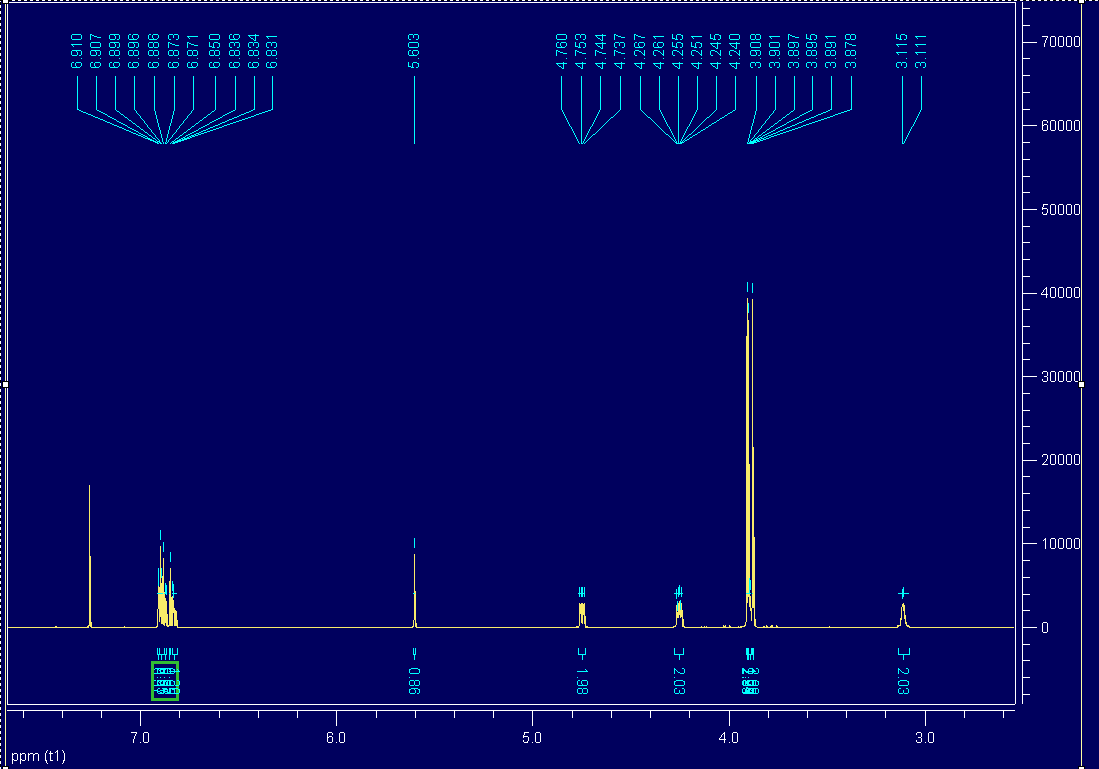


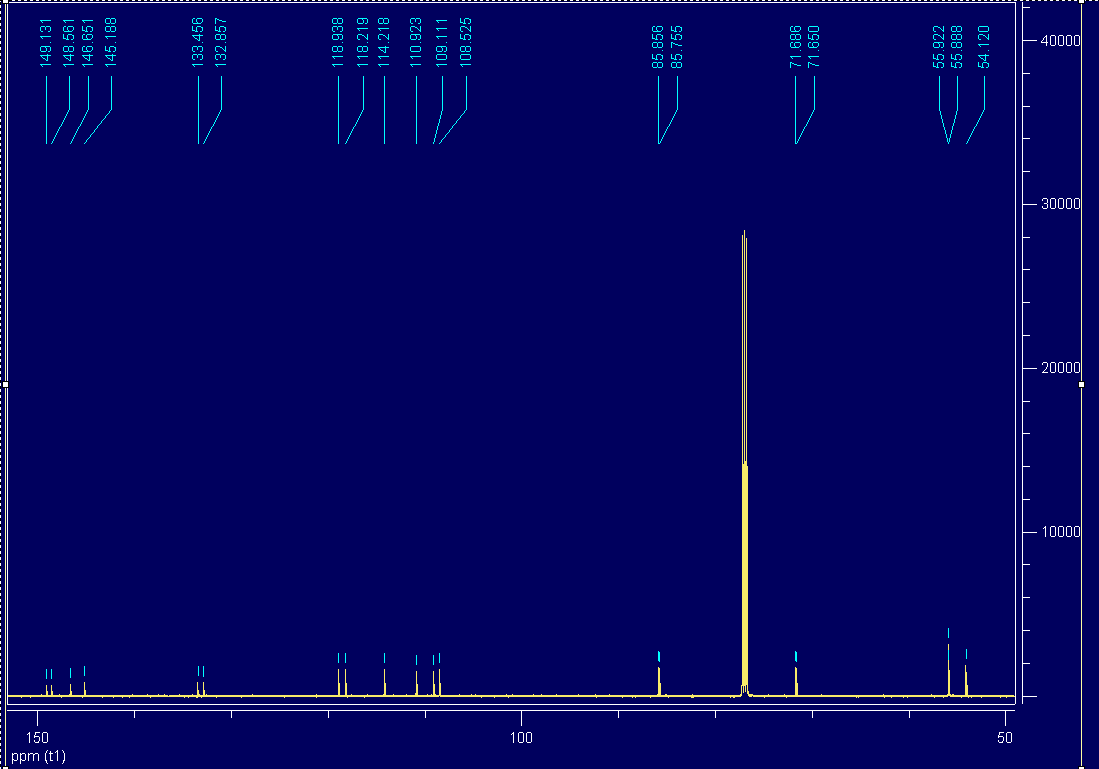


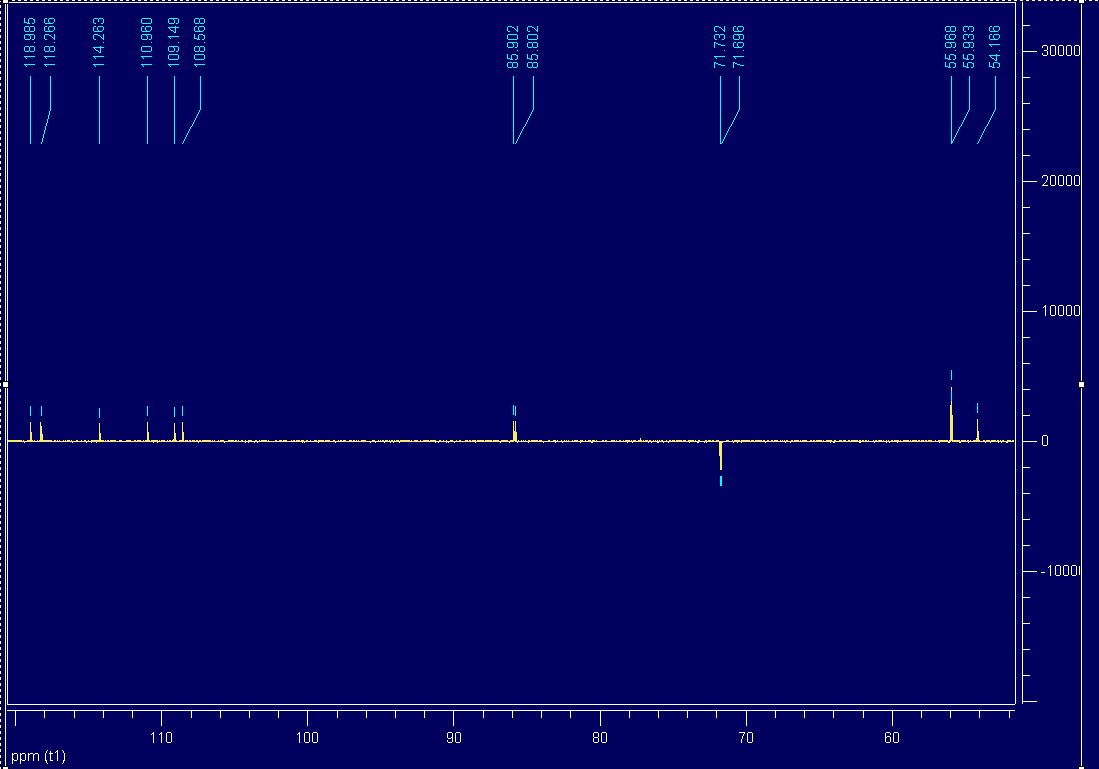


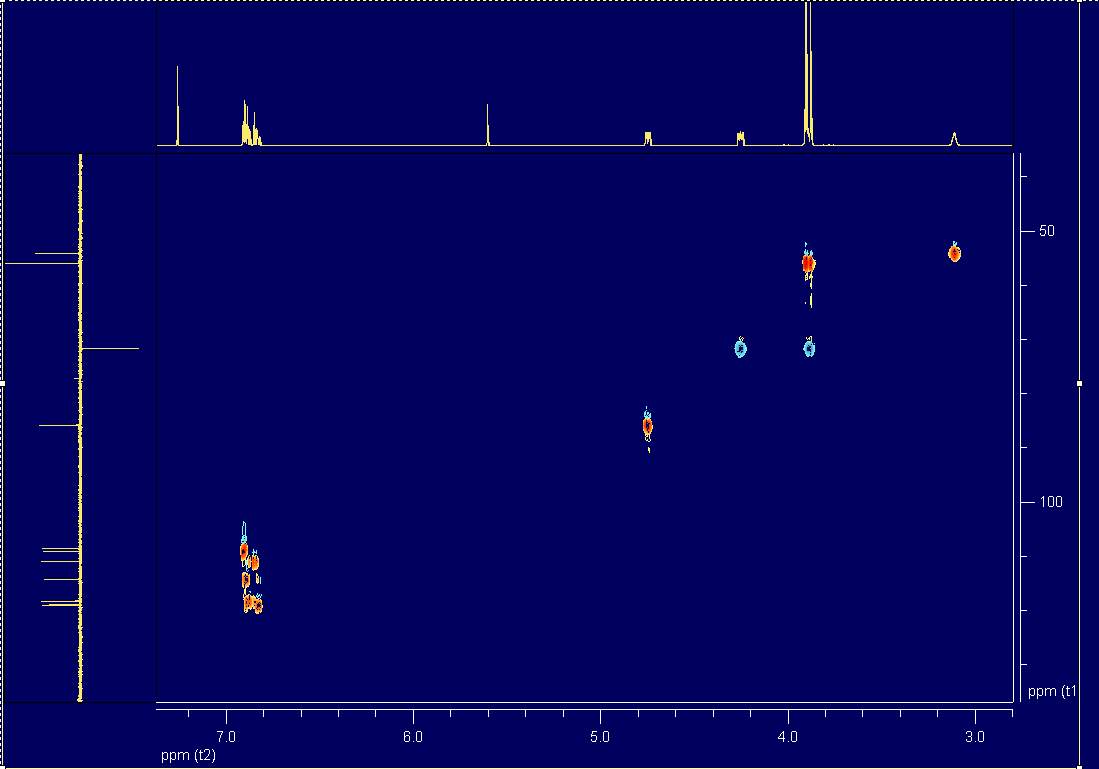


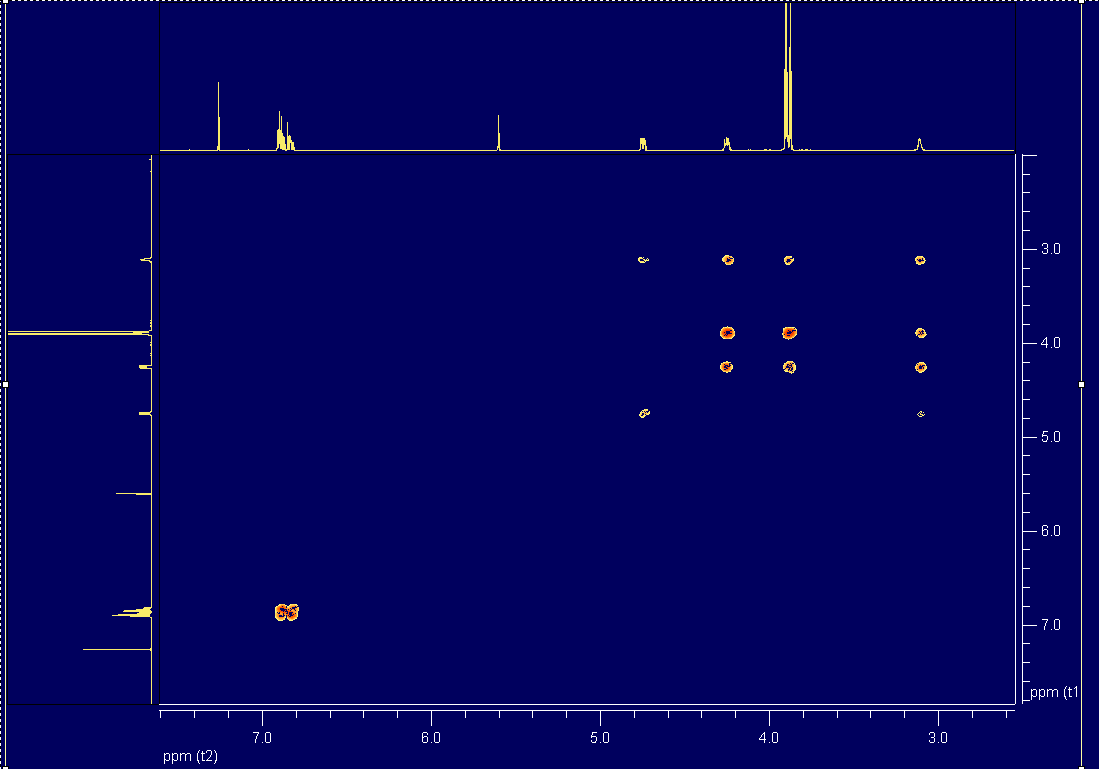

Supplement: S9 Fig — (DOC) [file pone.0126886.s009.doc]

**S10 Fig. 1H-NMR spectrum of mixtures of ursolic and oleanolic aldehydes 9 and 10 in CDCL3.**


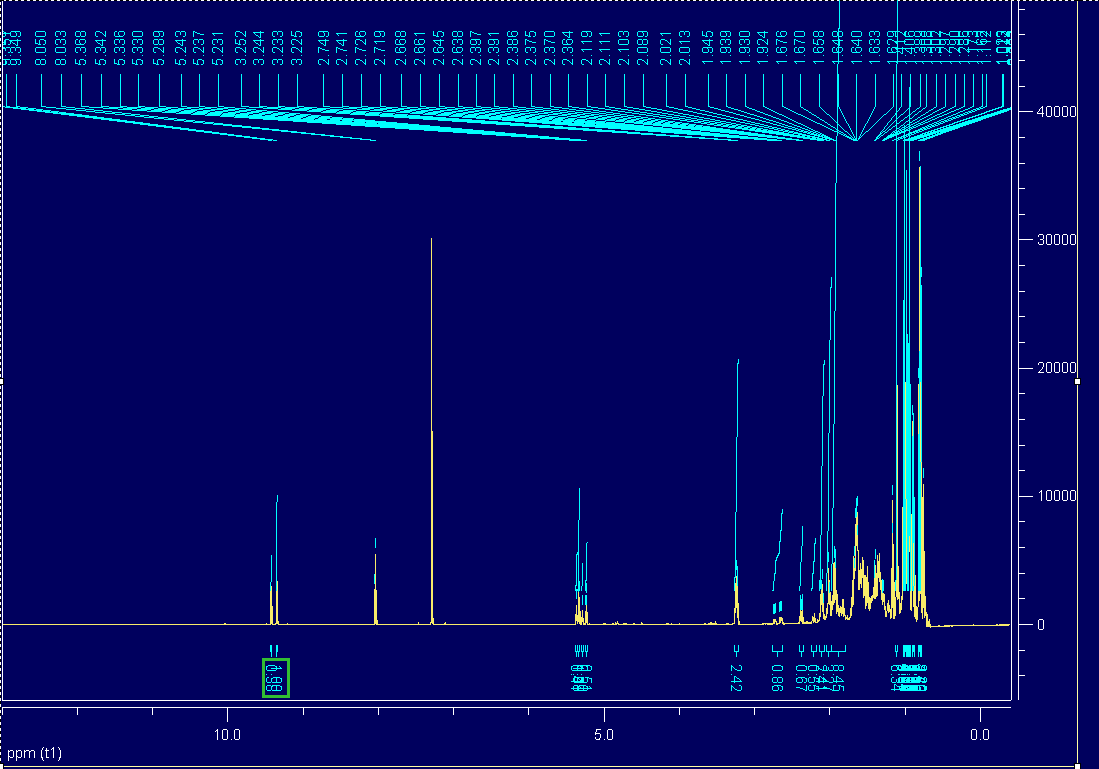

Supplement: S10 Fig — (DOC) [file pone.0126886.s010.doc]

**S11 Fig. 1H, 13C, DEPT, 1H-1H COSY, HSQC and HMBC NMR spectra of 3-*O*-acetyl ursolic acid 11 in CDCL3.**


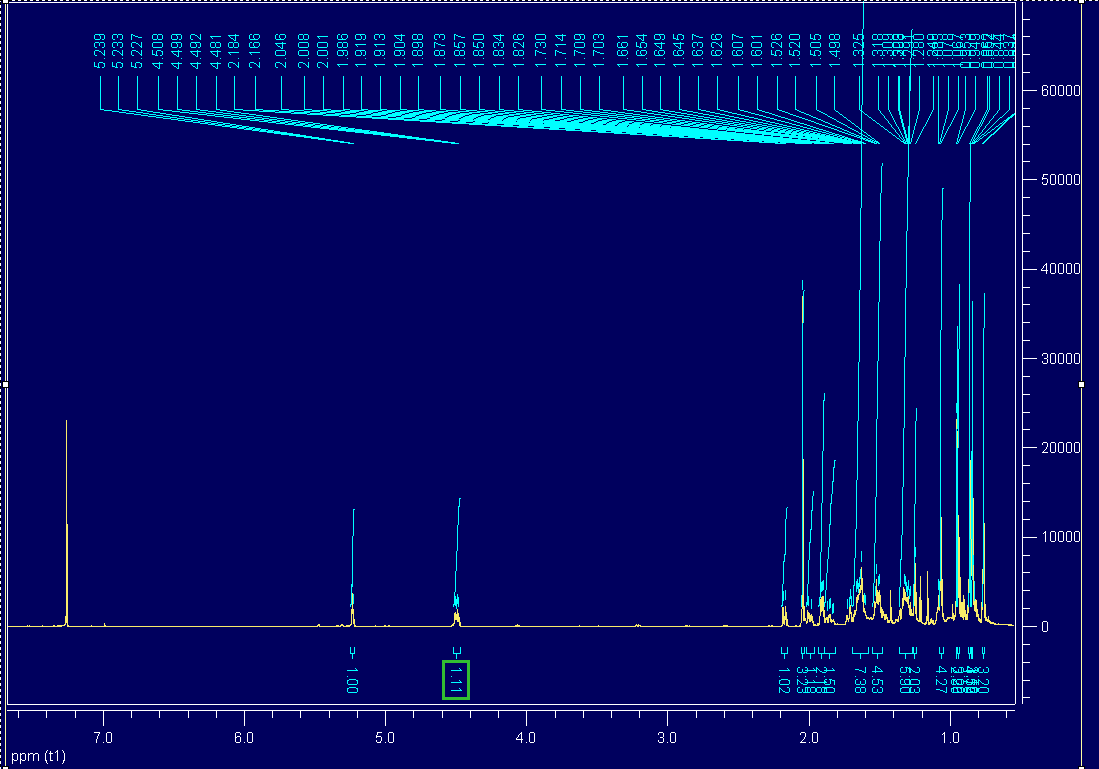


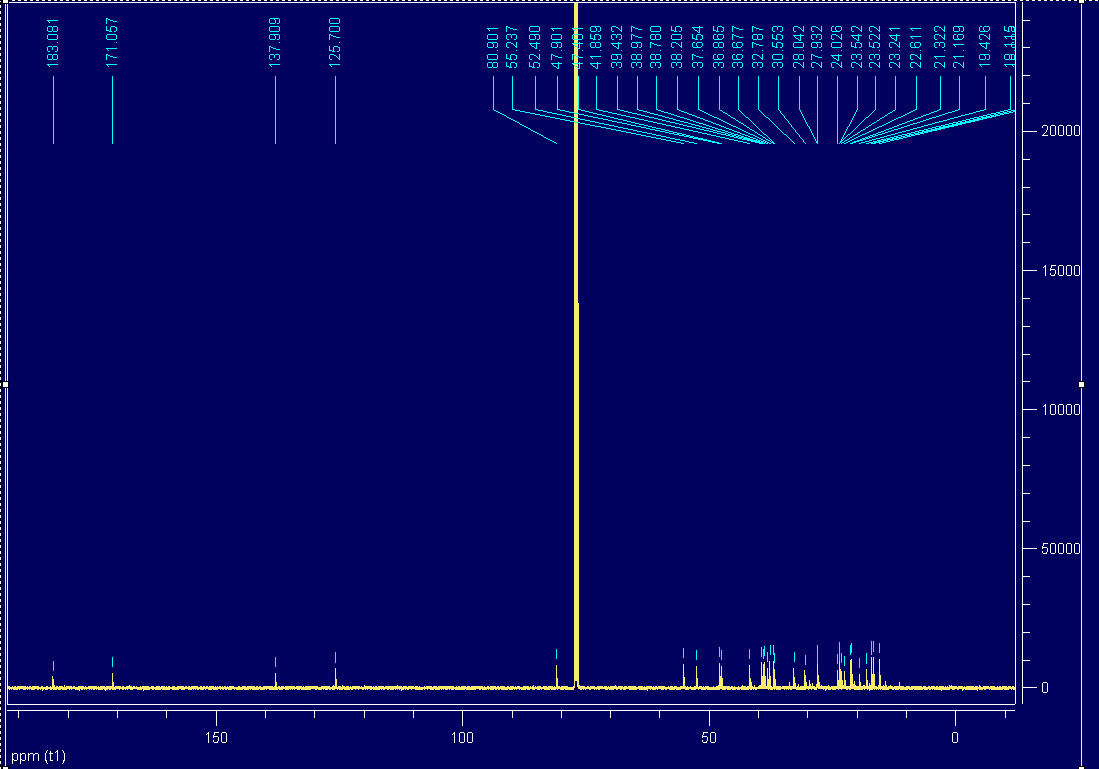


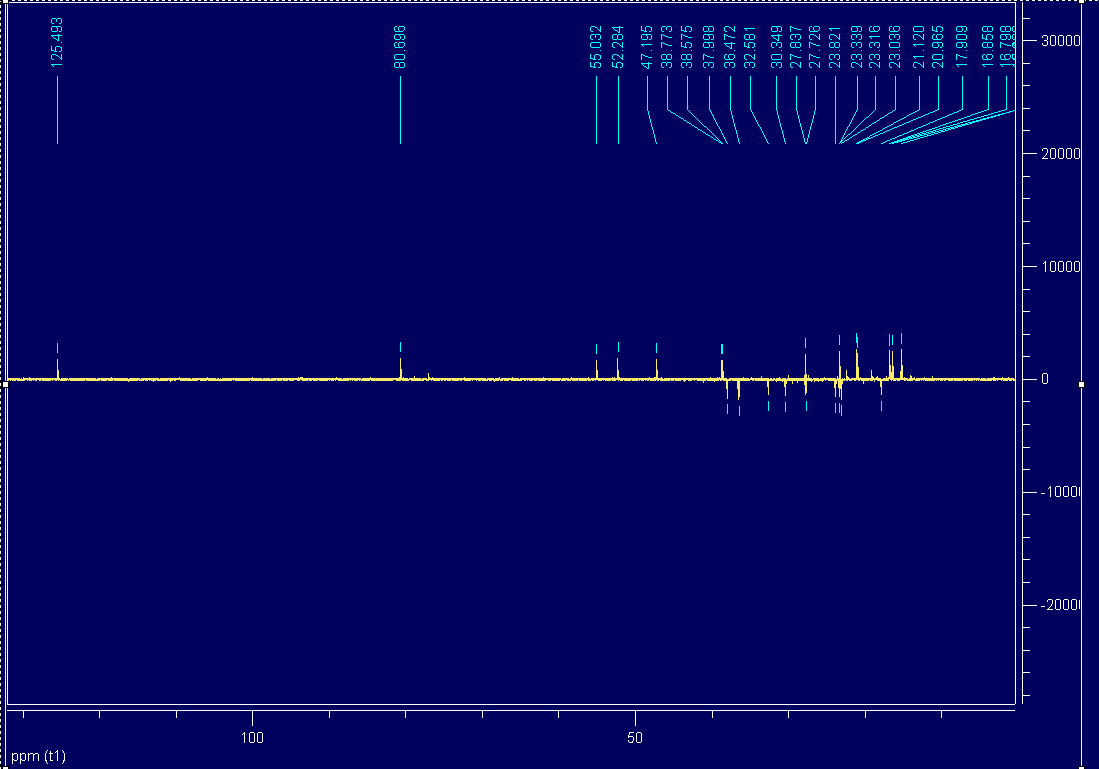


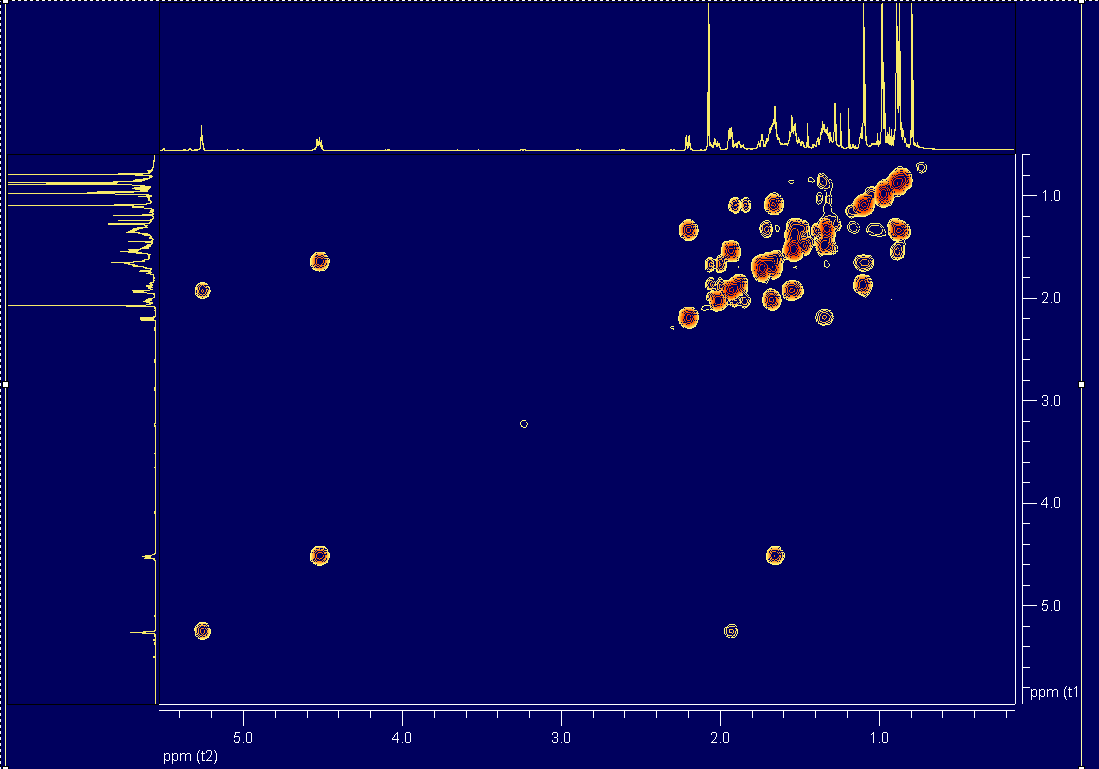


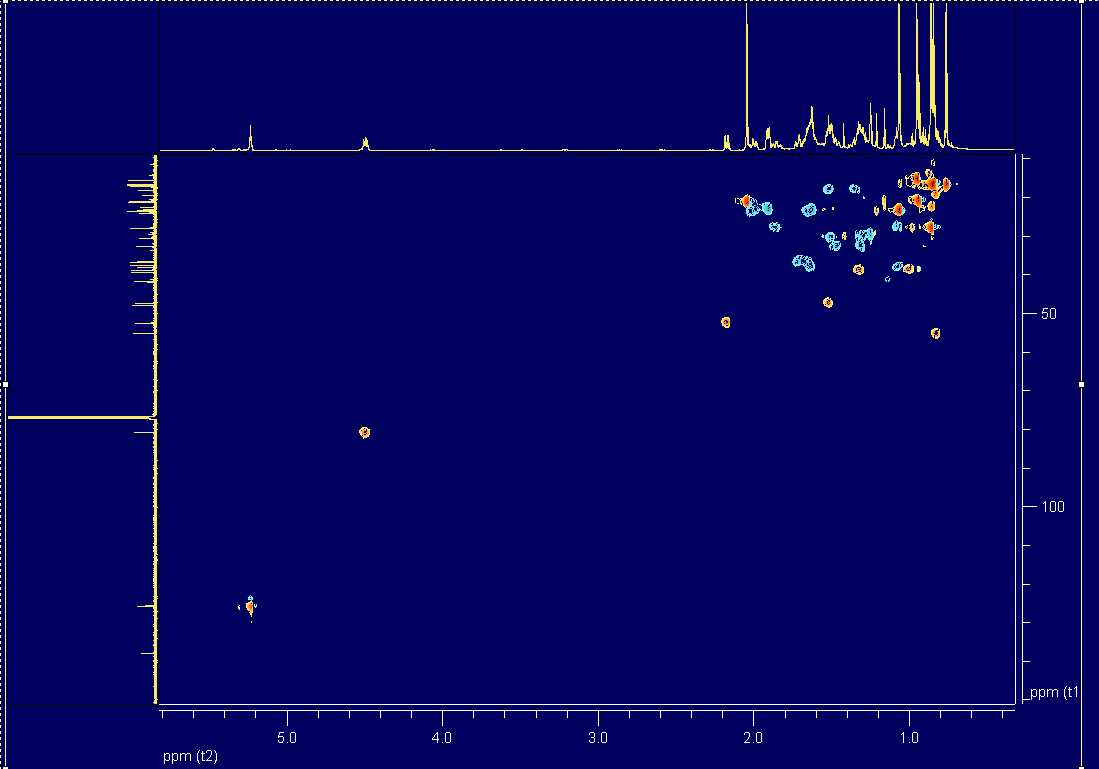


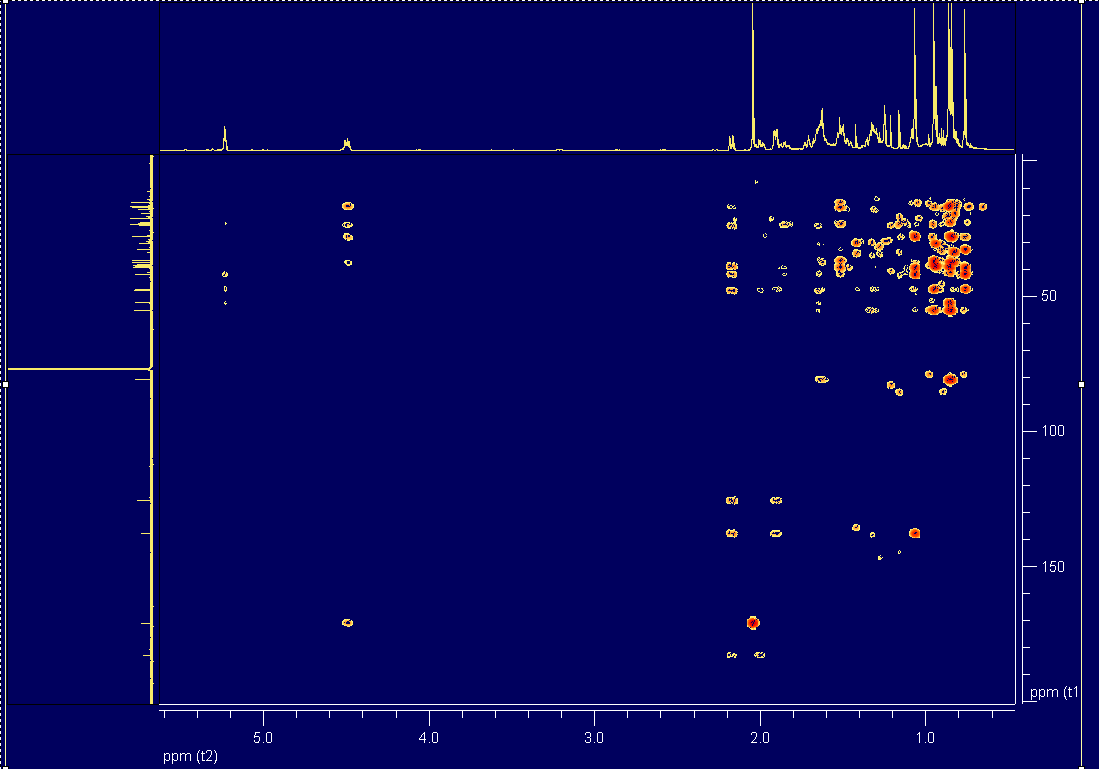

Supplement: S11 Fig — (DOC) [file pone.0126886.s011.doc]

**S12 Fig. 1H, 13C and DEPT NMR spectra of ocotillone I 12 in CD3OD:CDCL3 2:1.**


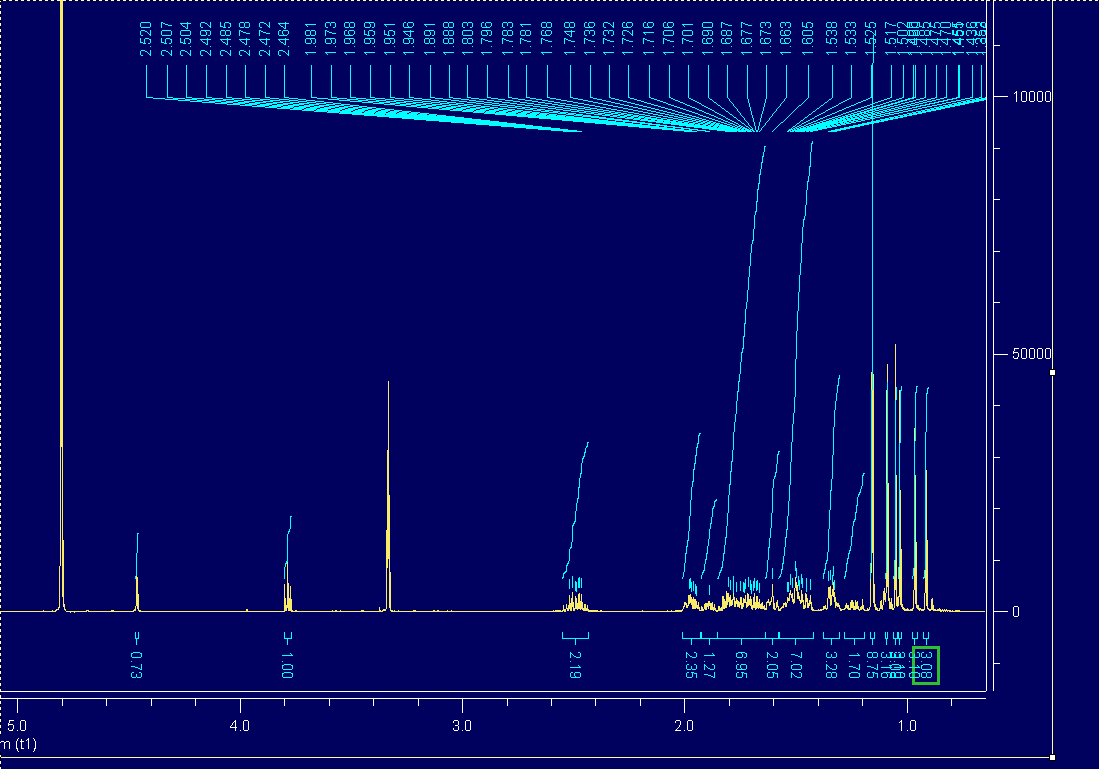


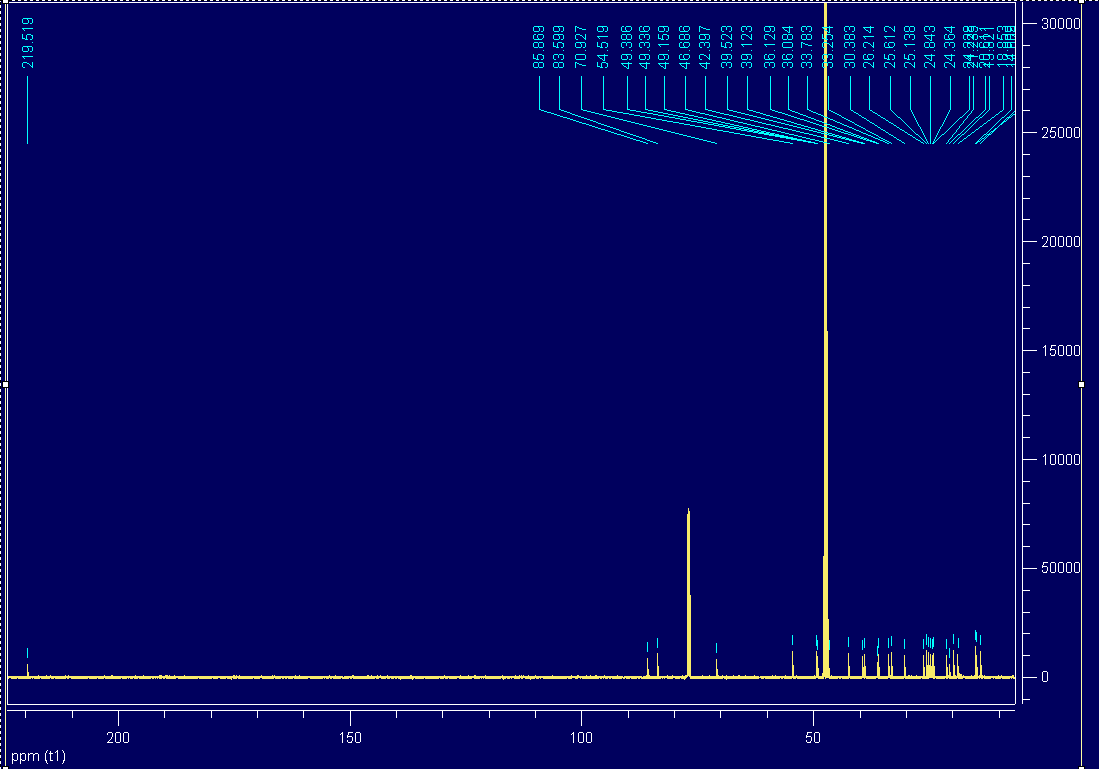


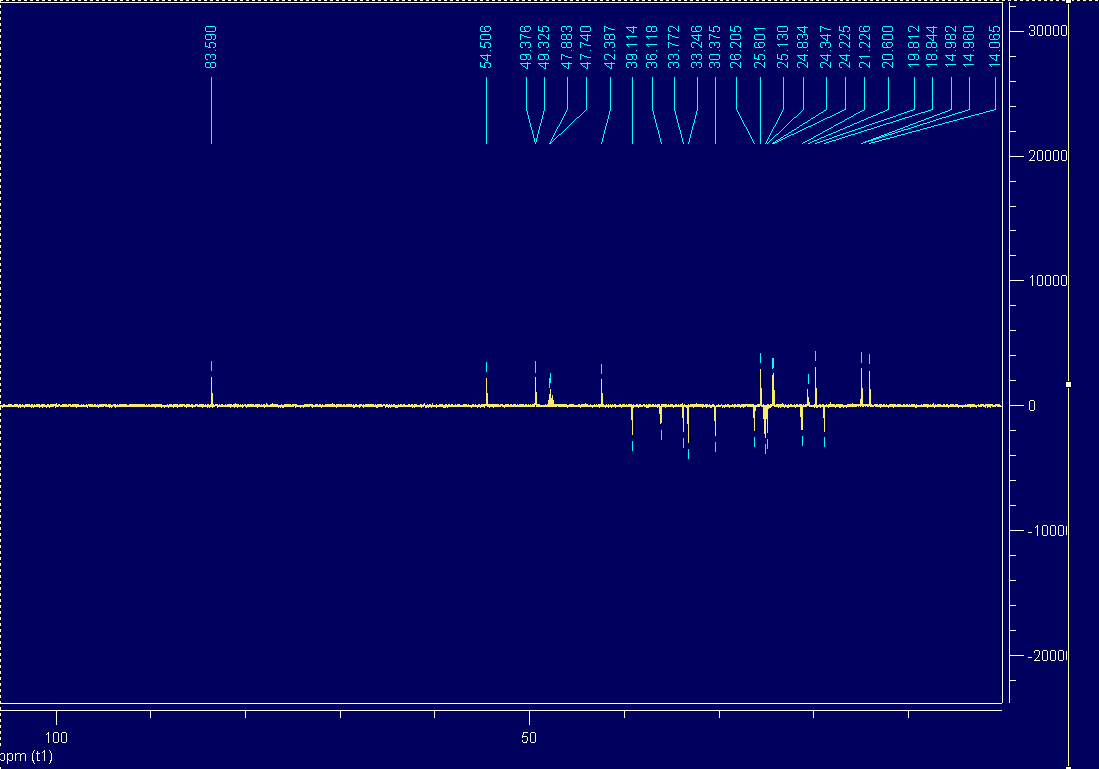

Supplement: S12 Fig — (DOC) [file pone.0126886.s012.doc]

**S13 Fig. 1H, 13C, DEPT, 1H-1H COSY, HSQC and HMBC NMR spectra of ocotillone II 13 in CD3OD:CDCL3 2:1.**


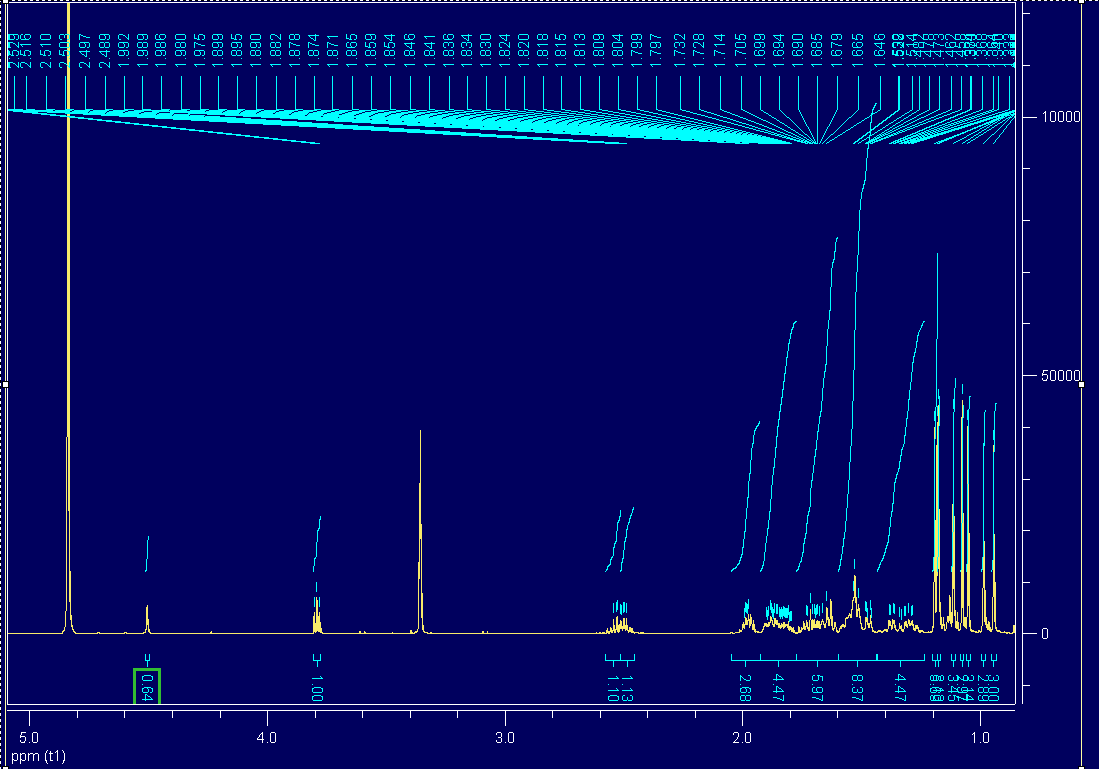


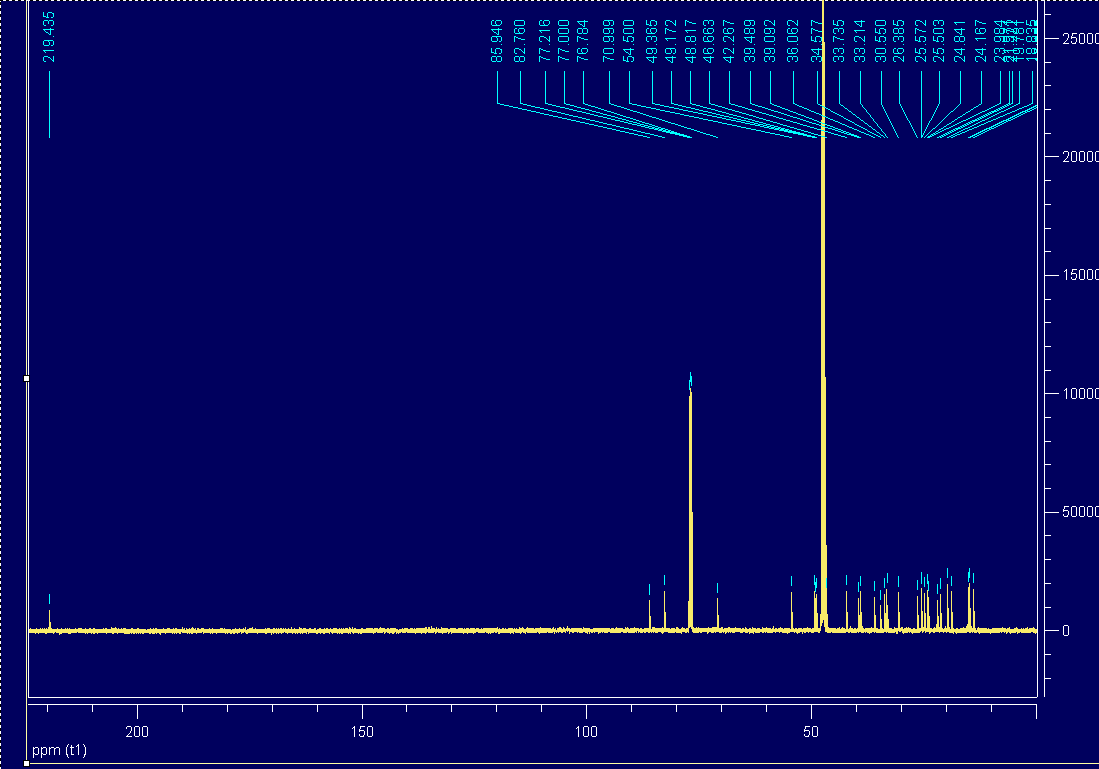


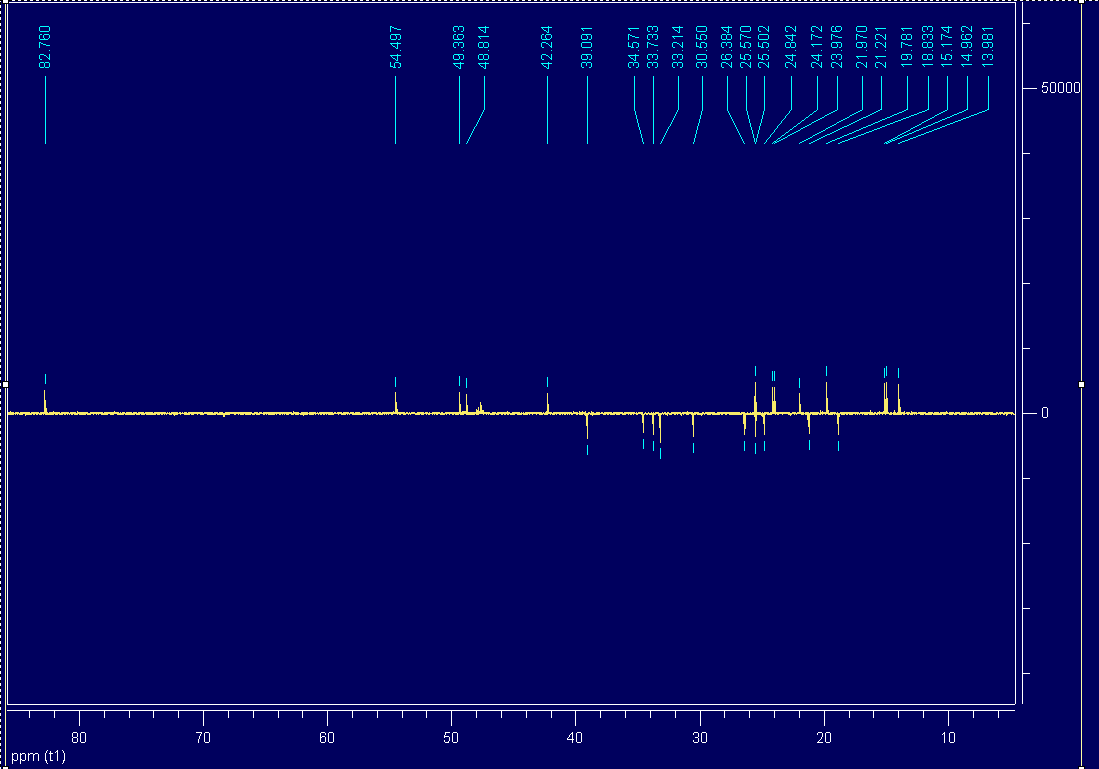


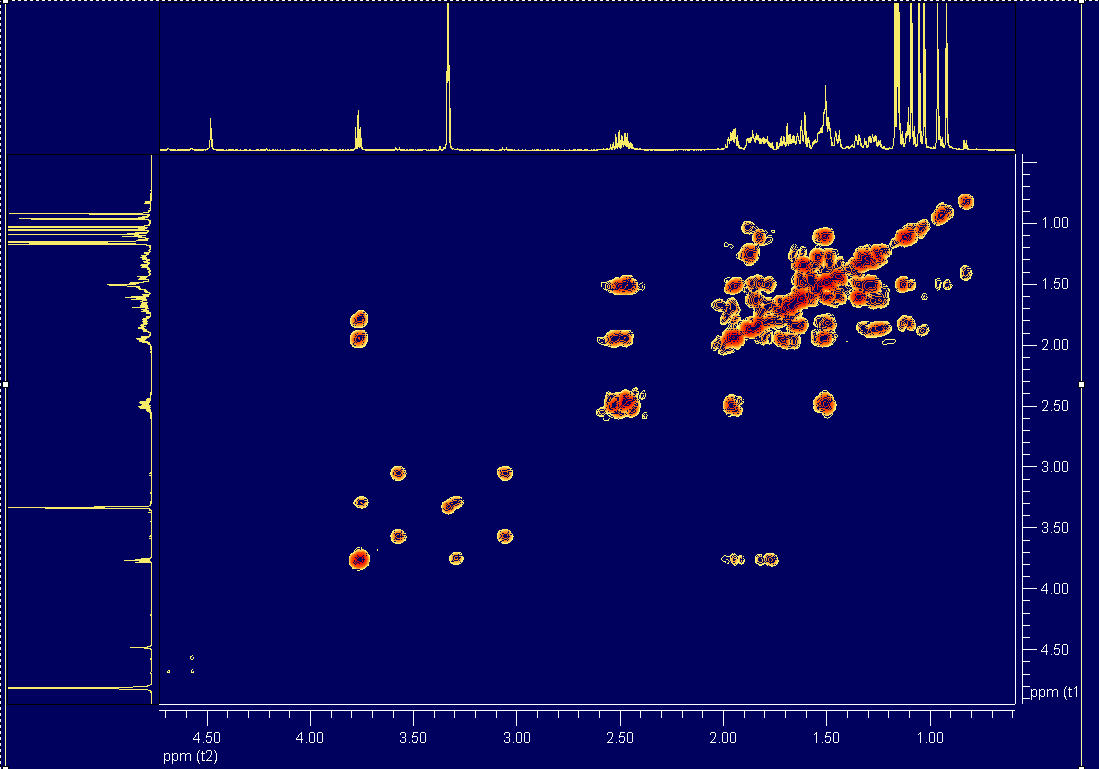


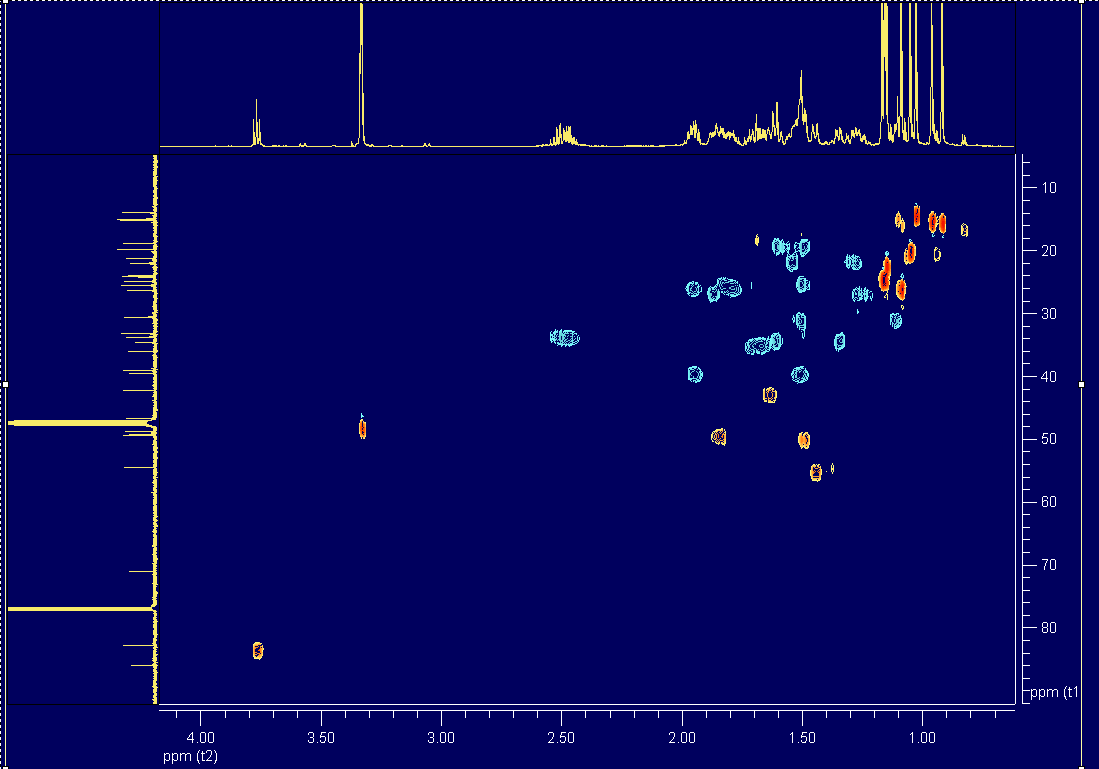


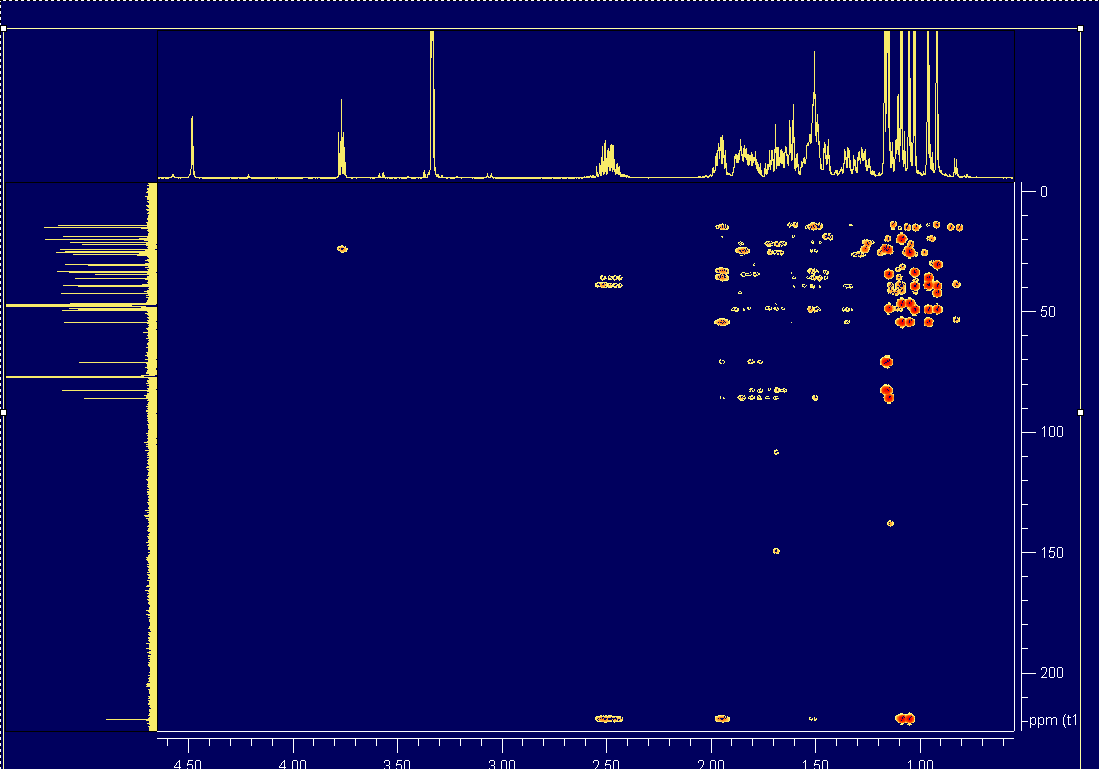

Supplement: S13 Fig — (DOC) [file pone.0126886.s013.doc]

**S14 Fig. 1H, 13C and DEPT NMR spectra of mixture of cabralealactones 14-15 in CD3OD:CDCL3 2:1.**


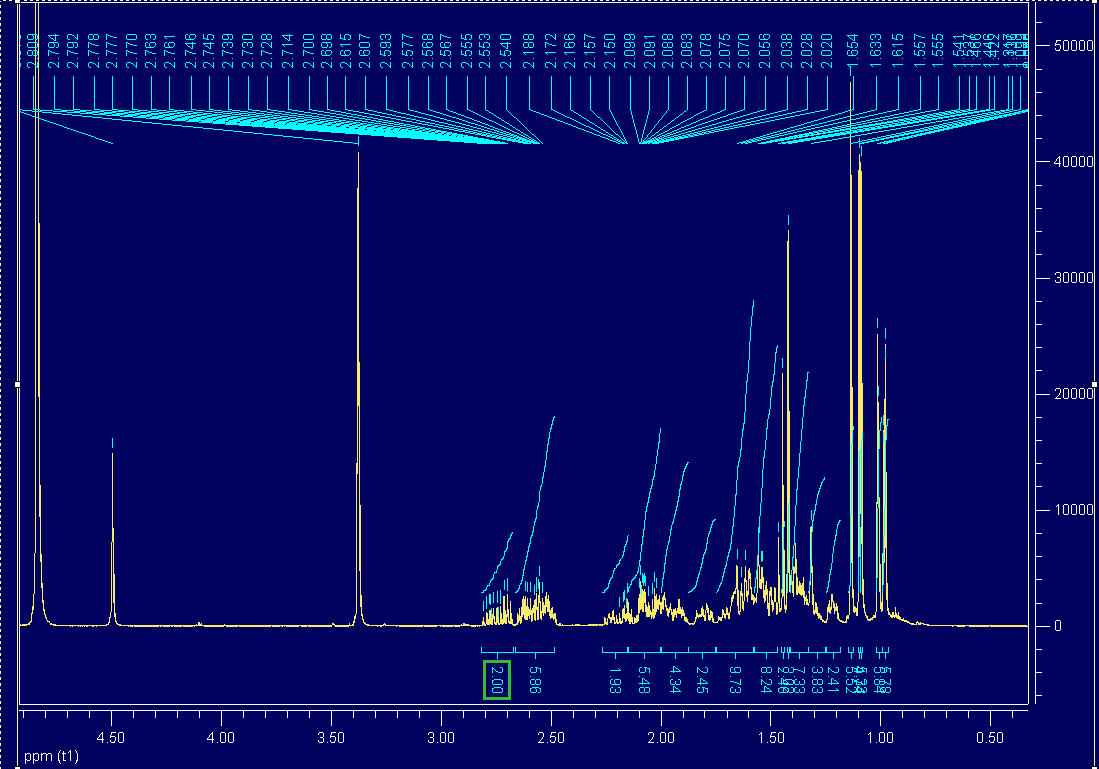


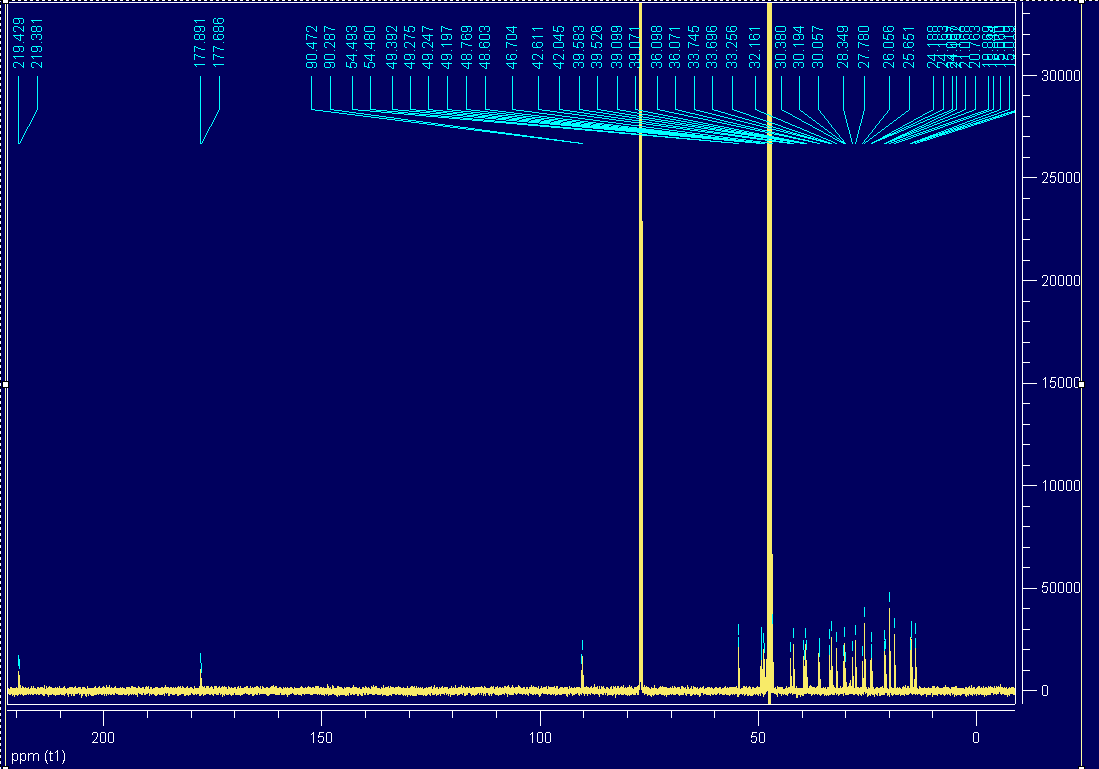


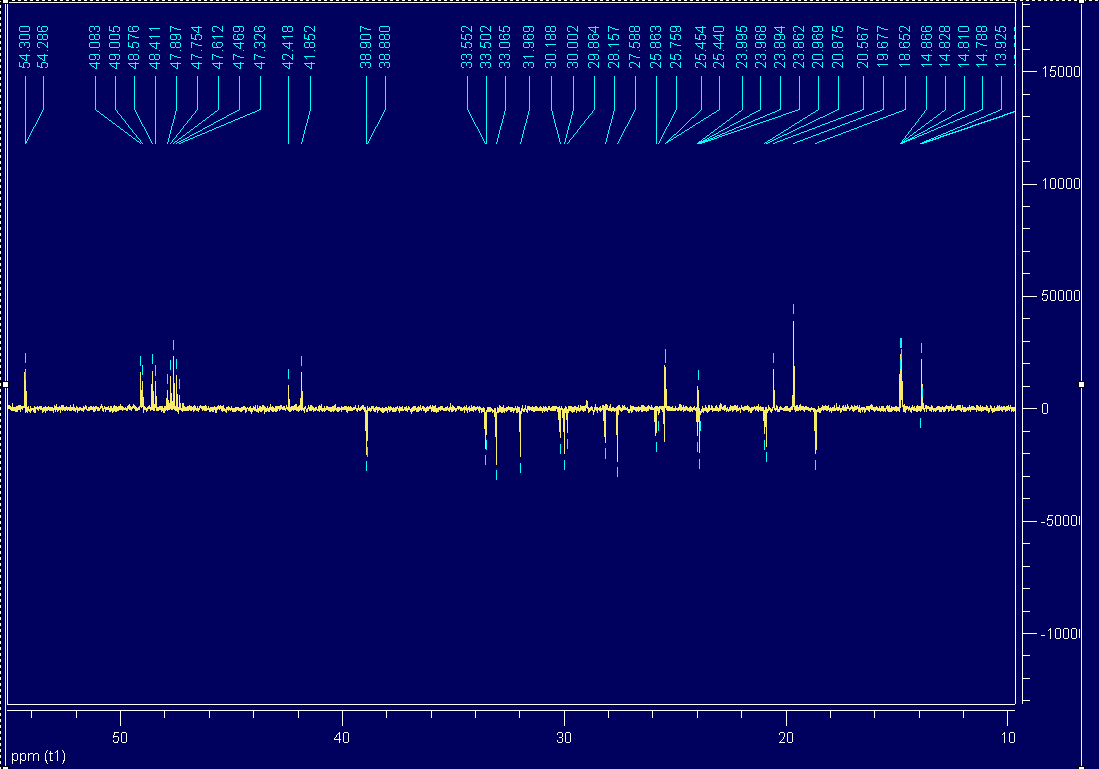

Supplement: S14 Fig — (DOC) [file pone.0126886.s014.doc]

**S15 Fig.** **Total ion chromatograms of silylated propolis ethanol extracts**


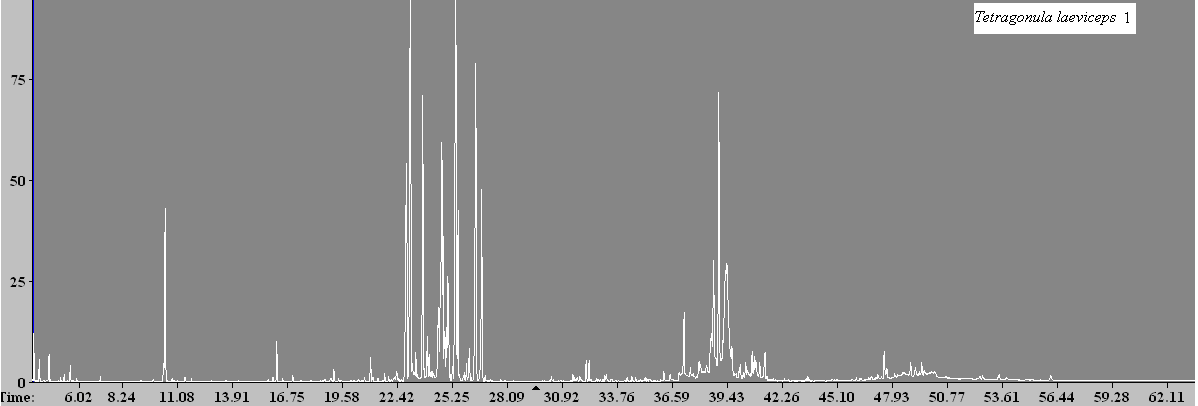


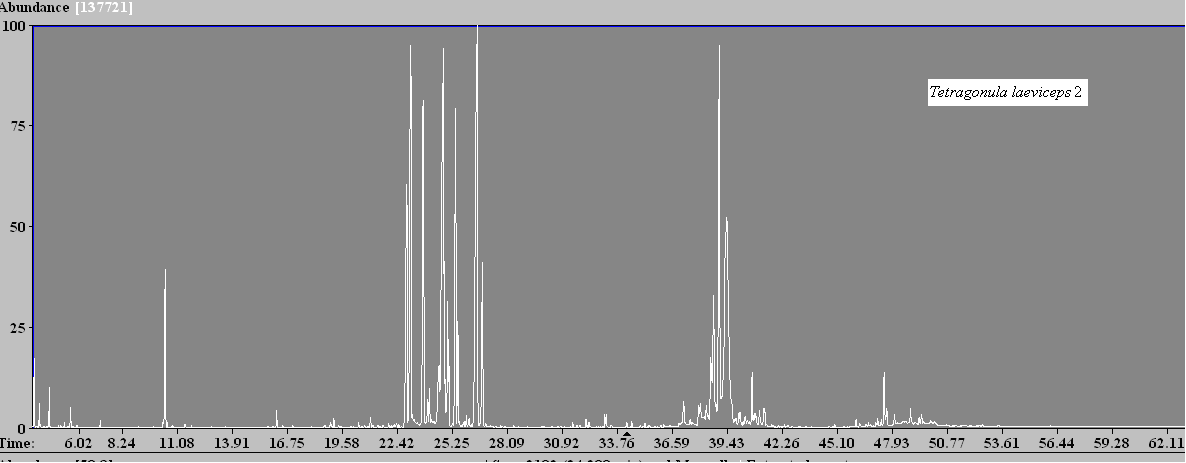


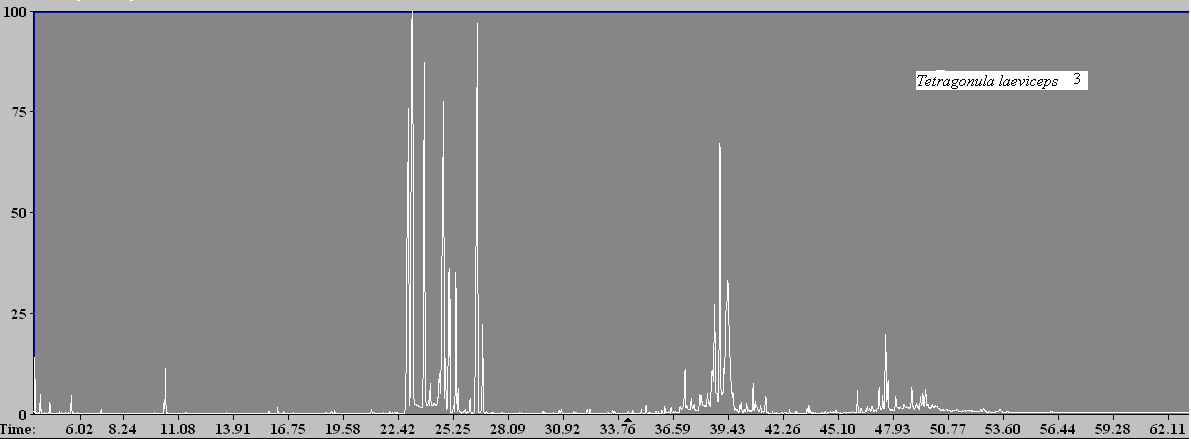


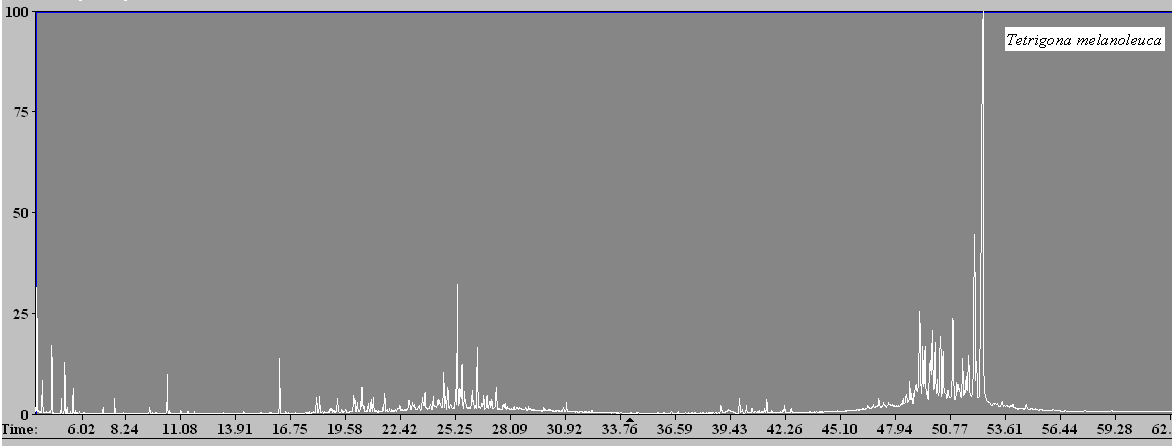

Supplement: S15 Fig — (DOC) [file pone.0126886.s015.doc]

**S16 Fig. Mass spectra of silylated maslinic and corosolic acids** (from GC-MS of sample *T. melanoleuca*)


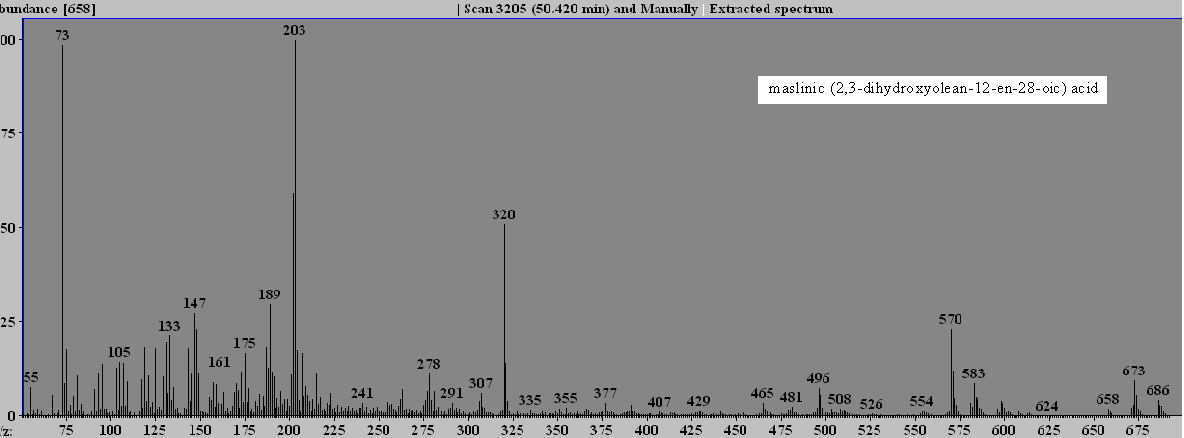


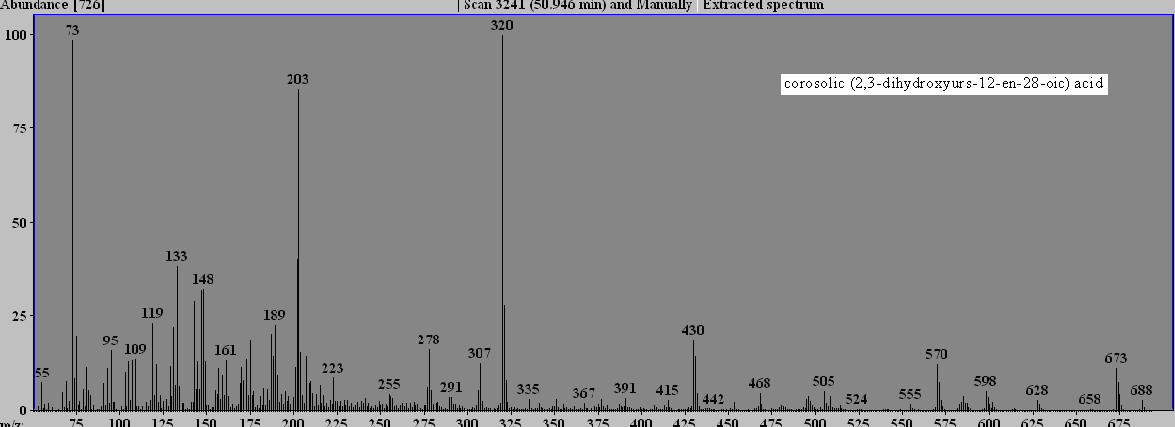

Supplement: S16 Fig — (DOC) [file pone.0126886.s016.doc]
