# Supplementary material for: Antibacterial Compounds from Propolis of Tetragonula laeviceps and Tetrigona melanoleuca (Hymenoptera: Apidae) from Thailand
Source: PLoS One. 2015 May 18;10(5):e0126886. doi: 10.1371/journal.pone.0126886 (PMC4436274; doi:10.1371/journal.pone.0126886)

**S17 Fig. Mass spectra of silylated 2,3-dihydroxyoleanadien-28-oic and 2,3-dihydroxyursadien-28-oic acids** (from GC-MS of sample *T. melanoleuca*)


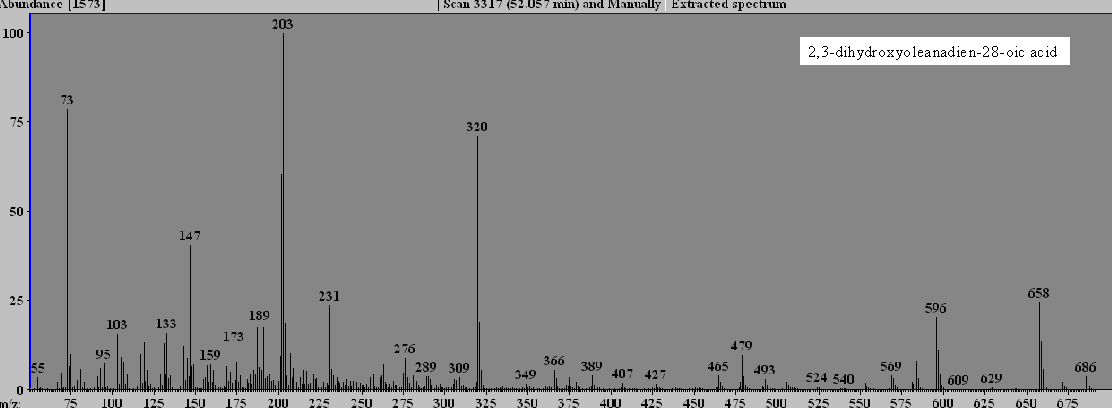


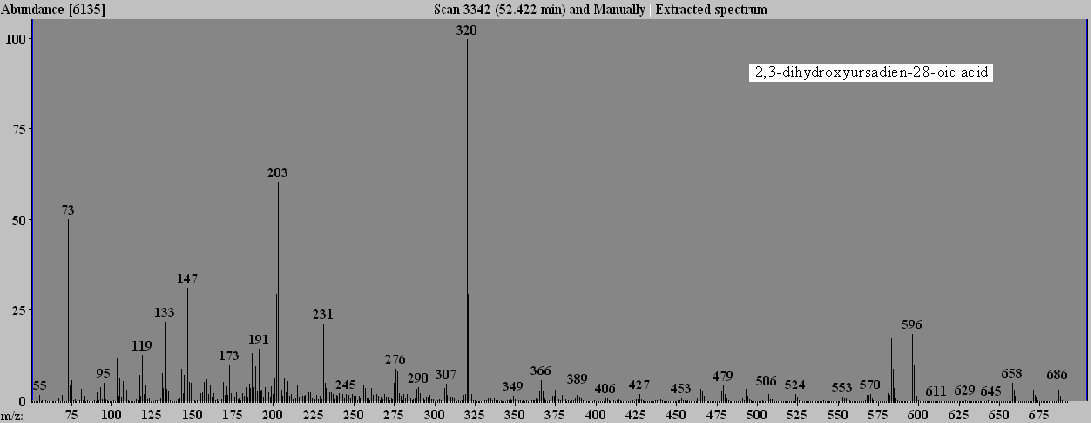

Supplement: S17 Fig — (DOC) [file pone.0126886.s017.doc]
